# Supplementary material for: Transition between conformational states of the TREK-1 K2P channel promoted by interaction with PIP2
Source: Biophys J. 2022 May 19;121(12):2380–8. doi: 10.1016/j.bpj.2022.05.019 (PMC9279171; doi:10.1016/j.bpj.2022.05.019)
Supplement: Document S1. Figures S1–S7 [file mmc1.pdf]

Biophysical Journal, Volume *121*

Supplemental information

Transition between conformational states of the TREK-1 K2P channel promoted by interaction with PIP_2

Adisorn Panasawatwong, Tanadet Pipatpolkai, and Stephen J. Tucker

Supplementary information

Number of molecules in each simulation system

Identification of PIP_2 binding site

Molecules	Up state	Down state
TREK-1	1	1
PC (upper leaflet)	252	252
PC (lower leaflet)	180	192
PS	36	38
PIP_2	24	25
CG-water	10968	11115
Na+	358	365
Cl-	198	198

Potential of mean force during the coarse-grained MD simulation

Molecules	Up state	Down state
TREK-1	1	1
PC (upper leaflet)	252	252
PC (lower leaflet)	236	253
PIP_2	1	1
CG-water	11192	11407
Na^+	207	207
Cl^-	198	198

Identification of PA binding site

Molecules	Up state	Down state
TREK-1	1	1
PC (upper leaflet)	252	252
PC (lower leaflet)	210	230
PA	23	25
CG-water	11458	11423
Na^+	248	252
Cl^-	198	198

Atomistic simulation of TREK-1 structure with PIP_2

Molecules	Up state	Down state
TREK-1	1	1
PC (upper leaflet)	250	250
PC (lower leaflet)	237	249
PIP_2	2	2
TIP3P	11615	11213
K^+	212	212
Cl^-	198	198

Atomistic simulation of TREK-1 structure without PIP_2

Molecules	Up state	Down state
TREK-1	1	1
PC (upper leaflet)	252	252
PC (lower leaflet)	234	256
TIP3P	11458	11471
K^+	202	202
Cl^-	198	198

Figure S1

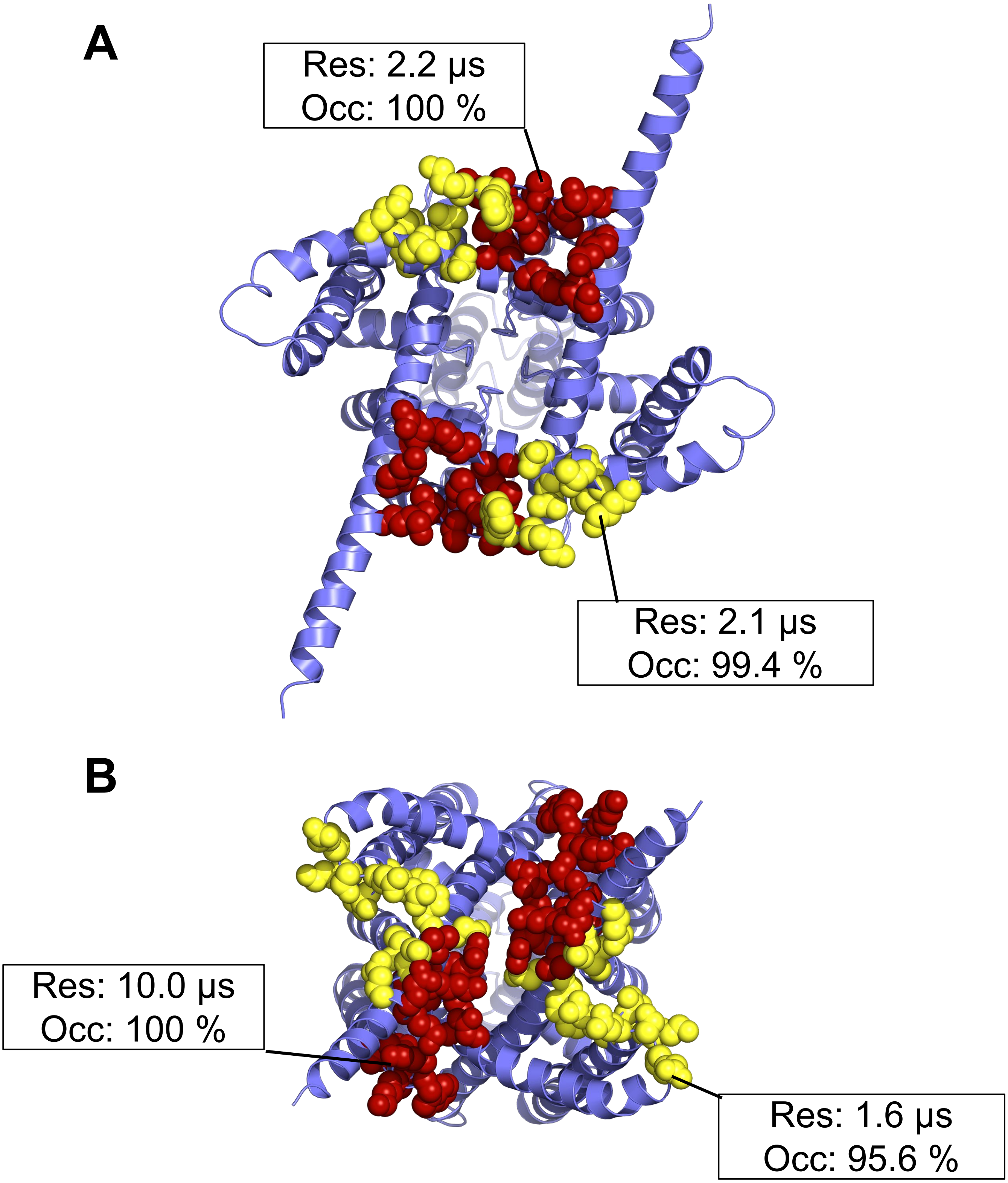

Figure S1. Bottom view of the PIP_2 binding sites from CG simulations

The PIP_2 binding site on the TREK-1 (A) up state or (B) down state. The binding site 1 (red) and 2 (yellow) are shown superimposed relative to their crystal structure. Each site is annotated with their residence time within the binding site (Res) and their occupancy (Occ).

Figure S2

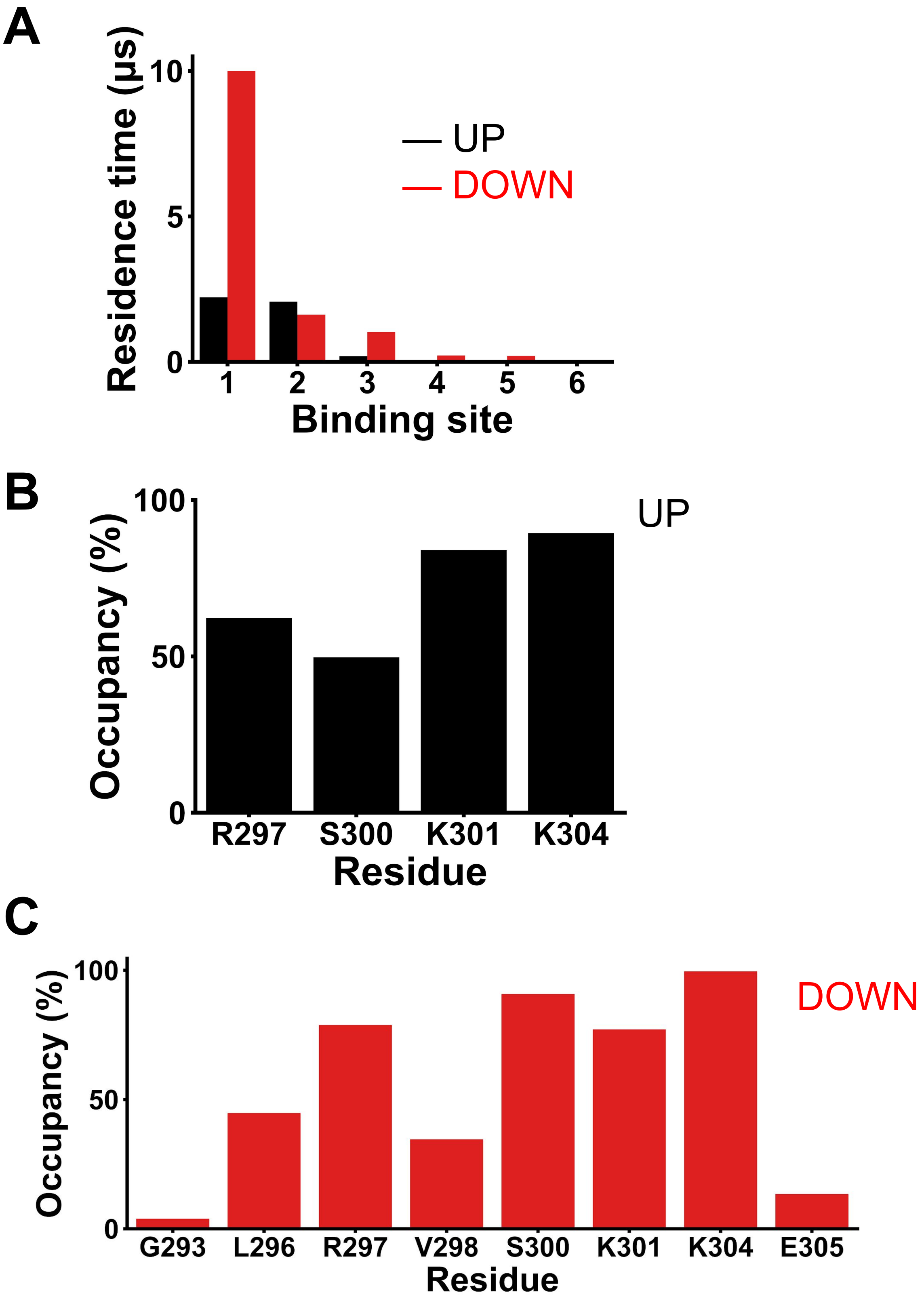

Figure S2. Residence time and occupancy of PIP_2 binding sites from CG simulations.

(**A**) The residence time of PIP_2 in binding sites in the up state (black) and down state (red), ordered by their residence time in the binding site. (**B**) Bar plot of occupancy of residue in the most prominent binding site in the up state (black) and down state (red) from 10 μs CG simulation.

Figure S3

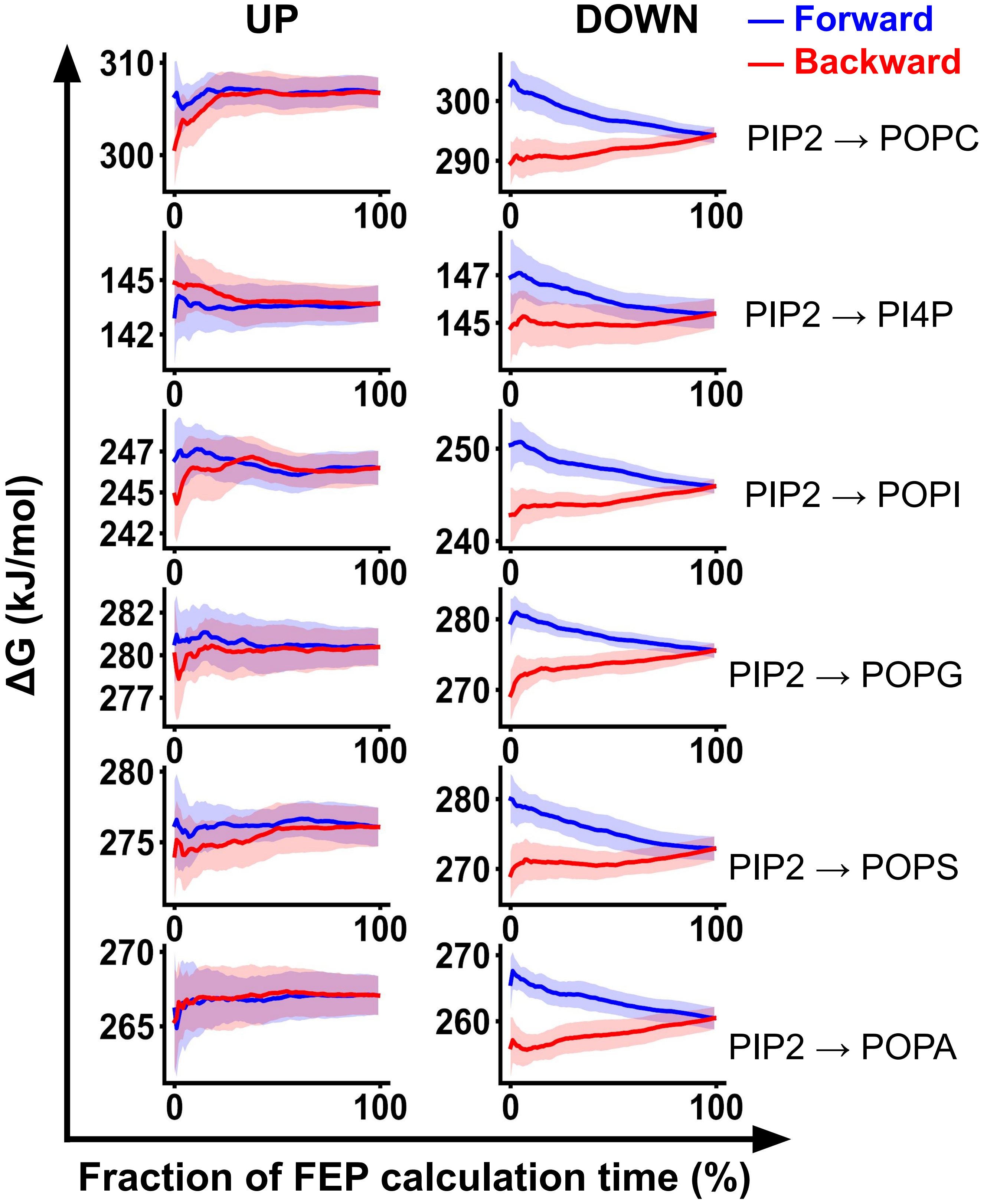

Figure S3. Hysteresis analysis of the free energy calculation whilst PIP_2 is being perturbed to different lipids

Free energy landscape where PIP_2 (0 % of the fraction of FEP calculation time) is perturbed to different lipids (100% of the fraction of the FEP calculation time). The free energy values were calculated from the forward (blue) or backward (red) during the perturbation process. Shaded region represents 95% confidence interval around the mean from 15 repeats.

Figure S4

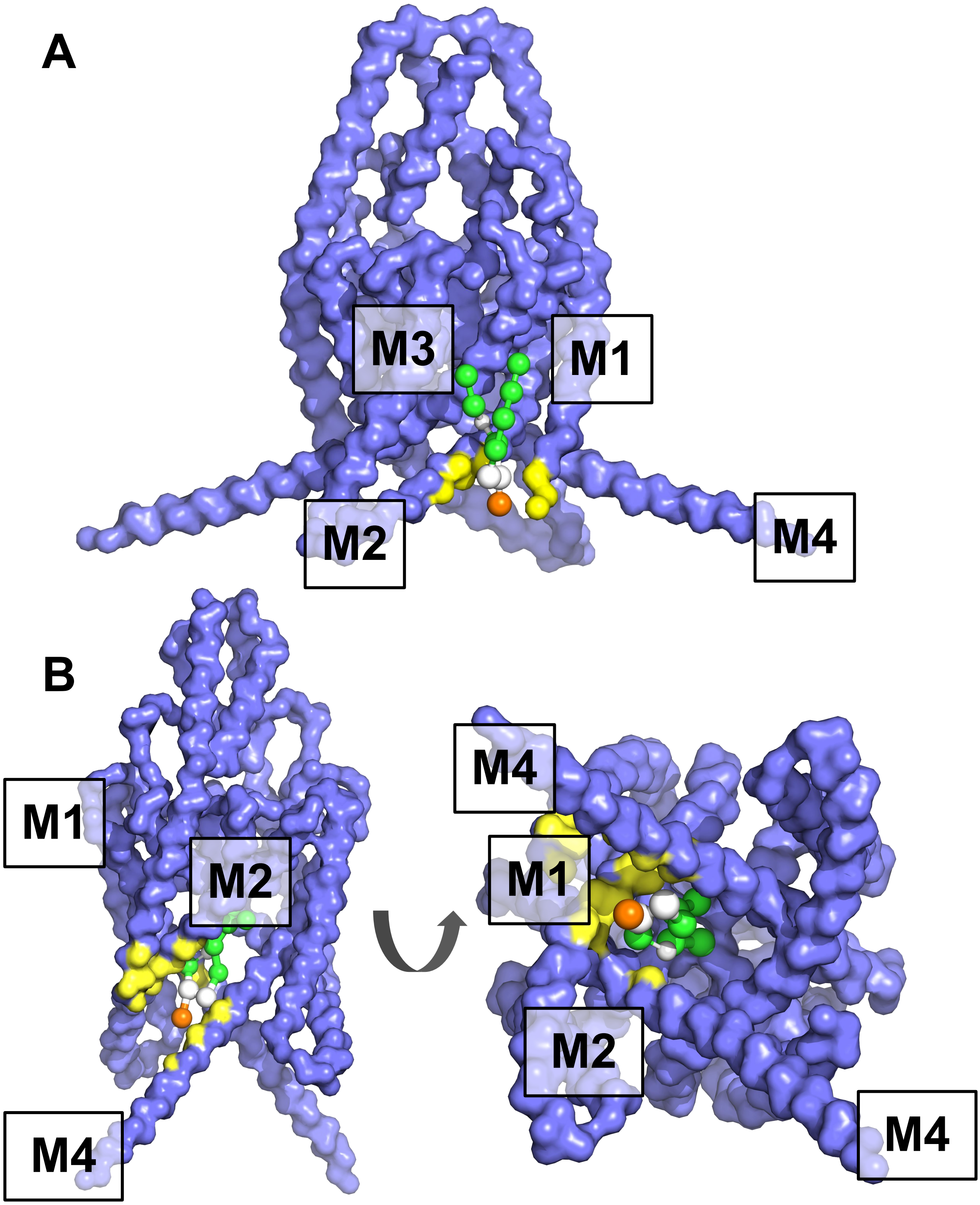

Figure S4. Phosphatidic acid (PA) binding site in the up and down state simulations.

The phosphatidic acid binding sites with highest occupancy and residence time (yellow) are shown superimposing with (**A**) up state and (**B**) down state crystal structures (blue) after a 10μs CG simulation.

Figure S5

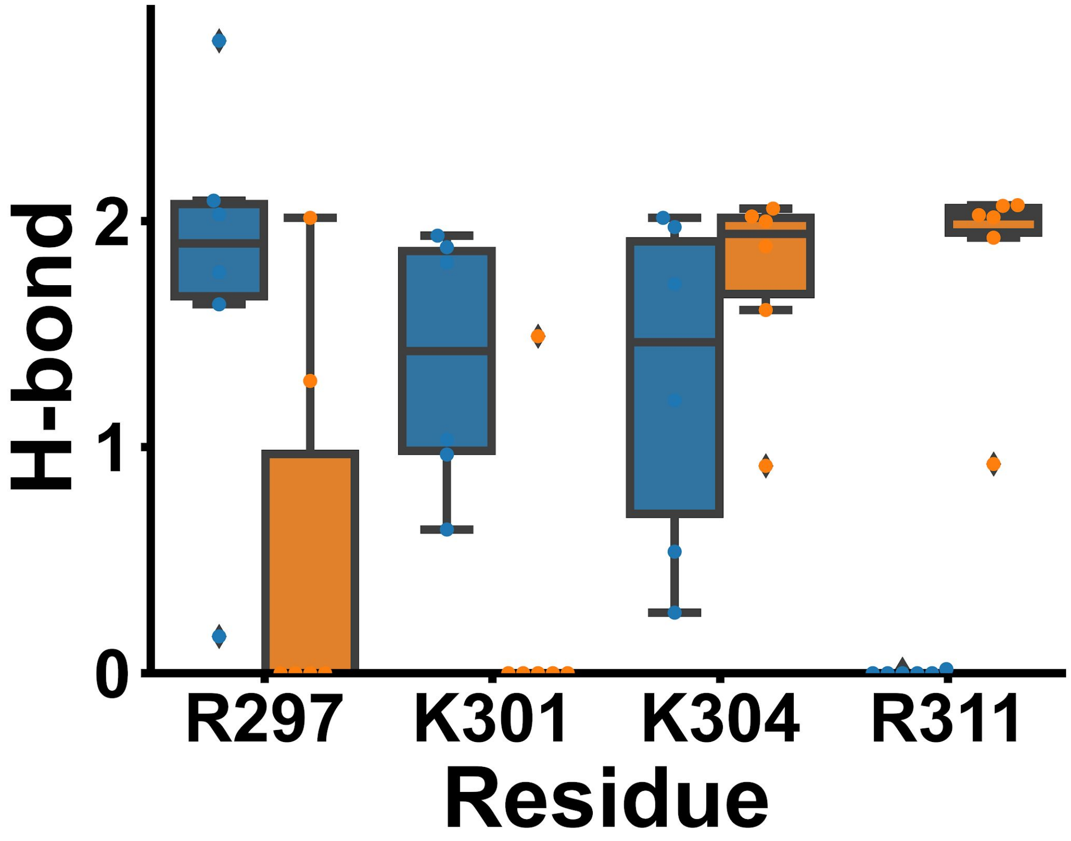

Figure S5. Hydrogen bonding between PIP_2 and TREK-1 in the up and down states

Median number of hydrogen bonds formed from the last 100 ns of the atomistic simulation of TREK-1 in the up state (blue) or down state (orange). Each plot contains six data points from three simulations and each subunit of the TREK-1 channel. The error bar represents the inter-quartile range of the distribution.

Figure S6

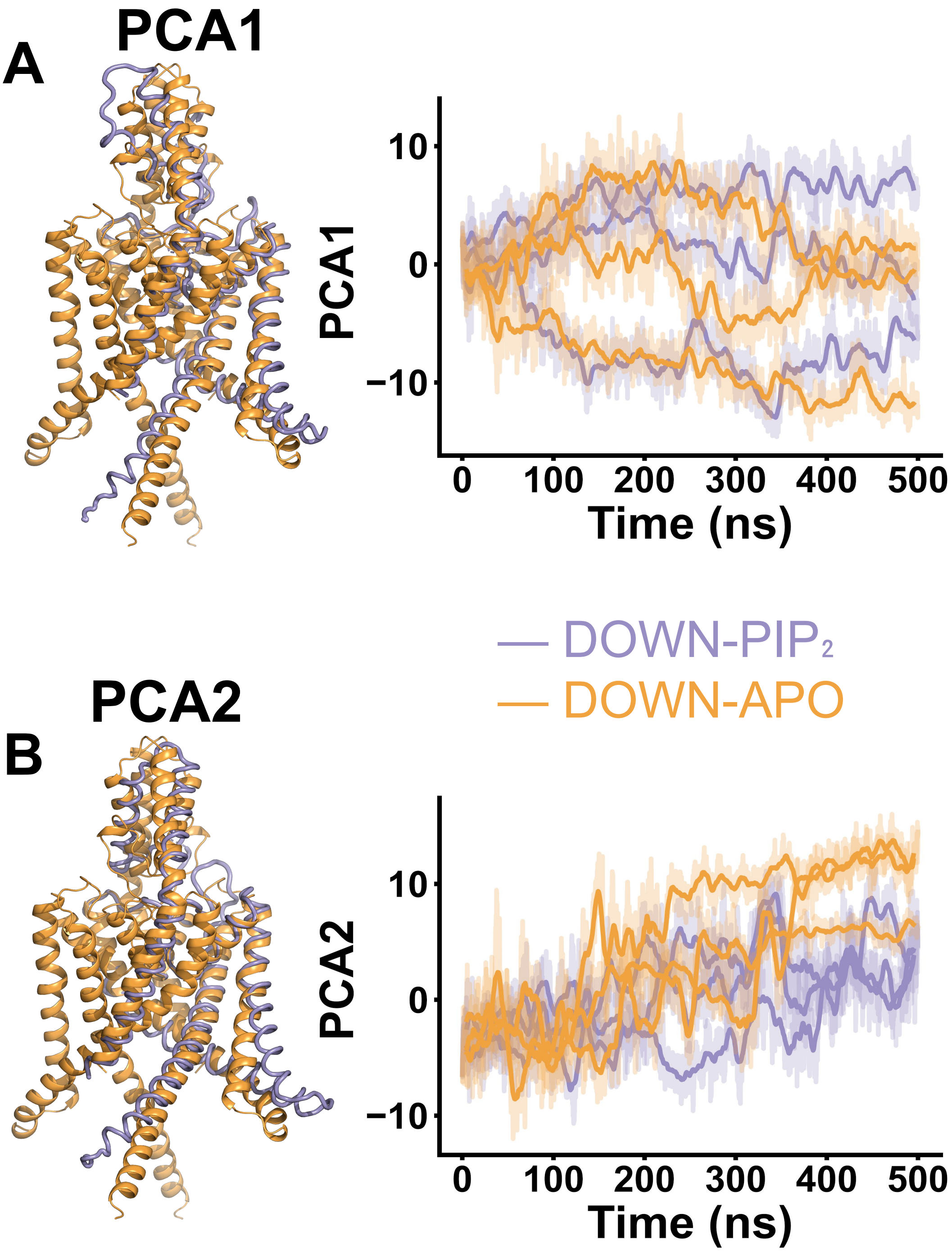

Figure S6. Principal component analysis of PIP2-induced movement in TREK-1

The principal components are calculated from three simulations of TREK-1 channel in the down state containing PIP_2. (**Left**) The backbone of the protein before the simulation (orange) is aligned to the interpolation of the of the most extreme projection along (**A**) the first principle and (**B**) the second principal component (purple) calculated from the simulation. (**Right**) The magnitude of the conformational change along the vector describing the first and second principal component derived from the simulation over 500 ns (n = 3). The simulations were conducted with PIP_2 (purple) and without PIP_2 (orange).

Figure S7

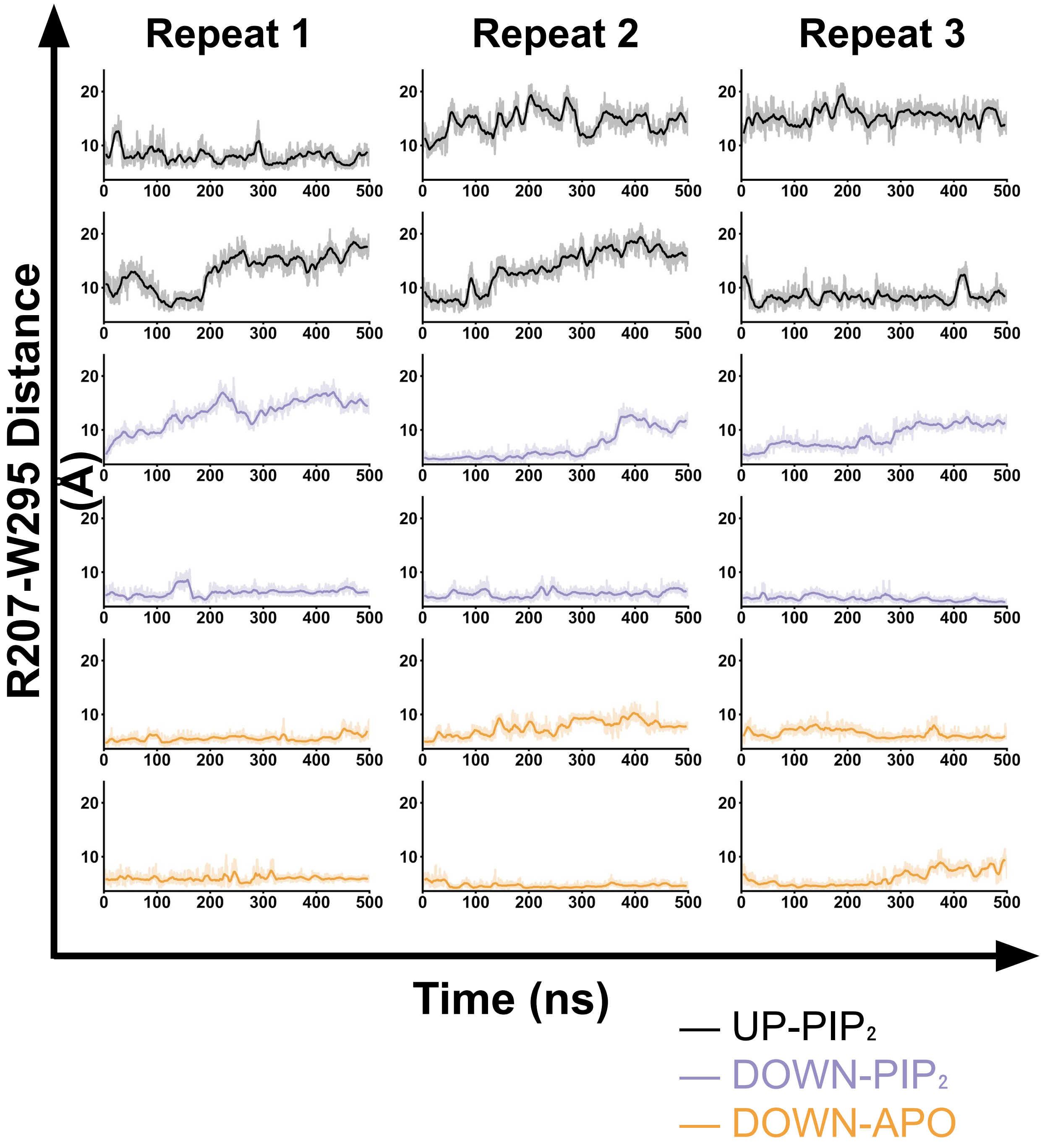

Figure S7. R207-W295 (zipper) distance in all simulations.

Distances between R207-W295 were calculated in three different simulation set-up (up state with PIP_2 - black, Down state with PIP_2 – purple, Down state without PIP_2 - orange). Each measurement represents the distance within each subunit of the TREK-1 channel.

Supplementary files

UP-Visual_Binding_sites.pse

TREK1 channel is represented as cartoon. Two binding sites with highest occupancy are represented in yellow and red spheres.

DOWN-Visual_Binding_sites.pse

TREK1 channel is represented as cartoon. Two binding sites with highest occupancy are represented in yellow and red spheres.
