# Supplementary material for: Transition between conformational states of the TREK-1 K2P channel promoted by interaction with PIP2
Source: Biophys J. 2022 May 19;121(12):2380–8. doi: 10.1016/j.bpj.2022.05.019 (PMC9279171; doi:10.1016/j.bpj.2022.05.019)
Supplement: Document S2. Article plus supporting material [file mmc4.pdf]

Biophysical Journal
Article

Biophysical Society

Transition between conformational states of the TREK-1 K2P channel promoted by interaction with PIP_2

Adisorn Panasawatwong,[1] Tanadet Pipatpolkai,[2,3,4,*] and Stephen J. Tucker[1,4,5,*]

[1]Department of Physics, University of Oxford, Oxford, United Kingdom; [2]Department of Physiology Anatomy and Genetics, University of Oxford, Oxford, United Kingdom; [3]Department of Biochemistry, University of Oxford, Oxford, United Kingdom; [4]OXION Initiative in Ion Channels and Disease, University of Oxford, Oxford, United Kingdom; and [5]Kavli Institute for Nanoscience Discovery, University of Oxford, Oxford, United Kingdom

ABSTRACT Members of the TREK family of two-pore domain potassium channels are highly sensitive to regulation by membrane lipids, including phosphatidylinositol-4,5-bisphosphate (PIP_2). Previous studies have demonstrated that PIP_2 increases TREK-1 channel activity; however, the mechanistic understanding of the conformational transitions induced by PIP_2 remain unclear. Here, we used coarse-grained molecular dynamics and atomistic molecular dynamics simulations to model the PIP_2-binding site on both the up and down state conformations of TREK-1. We also calculated the free energy of PIP_2 binding relative to other anionic phospholipids in both conformational states using potential of mean force and free-energy-perturbation calculations. Our results identify state-dependent binding of PIP_2 to sites involving the proximal C-terminus, and we show that PIP_2 promotes a conformational transition from a down state toward an intermediate that resembles the up state. These results are consistent with functional data for PIP_2 regulation, and together provide evidence for a structural mechanism of TREK-1 channel activation by phosphoinositides.

SIGNIFICANCE TREK-1 channels are involved in many vital physiological processes. Transitions between different conformational states of the channel influence their activity and hence affect cellular electrical excitability. Here, we use coarse-grained and atomistic molecular dynamics simulations to demonstrate that transition from the down to the up state can be promoted by interaction with the important membrane lipid phosphatidylinositol-4,5-bisphosphate. These simulations therefore propose a simple molecular mechanism for how phosphoinositides such as phosphatidylinositol-4,5-bisphosphate can directly modulate TREK channel activity.

INTRODUCTION

The TREK subfamily of two-pore domain K^+ channels contribute to the resting membrane potential and electrical activity of a wide variety of cell types and tissues including many within the central and peripheral nervous systems (1). They are involved in processes such as pain perception, neuroprotection, and anesthesia (2) and therefore make attractive therapeutic targets (3).

TREK channel activity is regulated by many different physical and chemical stimuli; this polymodal regulation allows them to integrate cellular electrical activity with a diverse range of cellular signaling pathways. In particular, TREK channels are mechanosensitive and highly sensitive to their lipid-membrane environment. Previous studies have shown that membrane tension increases channel activity by expanding the cross-sectional volume of the channel within the bilayer, thereby rendering it mechanosensitive (4). In addition to the physical properties of the membrane, TREK channels are also highly sensitive to different lipid species as well as both internal and external pH (5,6). For example, amino acids in the C-terminal domain (CTD) are involved in phosphatidylinositol-4,5-bisphosphate (PIP_2) regulation and intracellular pH sensing (7), while the CTD

Submitted March 17, 2022, and accepted for publication May 16, 2022.

*Correspondence: tanadet.pipatpolkai@scilifelab.se or stephen.tucker@physics.ox.ac.uk

Adisorn Panasawatwong's present address is Max Planck Institute for the Physics of Complex System, Dresden, Germany.

Tanadet Pipatpolkai's present address is Science for Life Laboratory, Department of Applied Physics, KTH Royal Institute of Technology, Solna, Sweden.

Editor: Sudha Chakrapani.

https://doi.org/10.1016/j.bpj.2022.05.019

© 2022 Biophysical Society.

This is an open access article under the CC BY license (http://creativecommons.org/licenses/by/4.0/).

also provides binding sites for proteins that can regulate lipid composition such as phospholipase D2 (8,9). However, despite the obvious importance of its lipid sensitivity and PIP_2 regulation in particular, relatively little is known about how PIP_2 interacts with TREK channels.

The many different crystal structures of TREK channels that are now available can be grouped into two main conformational states: an "up" state (e.g., PDB: 6CQ6) and a "down" state (e.g., PDB: 4XDJ) (10,11). The up state has a broader cross-section than the down state within the lower leaflet due to an upwards movement of the M2 and M4 transmembrane helices. However, the structure of the full CTD remains unknown, as this domain is truncated in all of the constructs used for crystallization. Both conformations of the channel appear to be conductive (12,13), but several studies indicate that the channel becomes more active when it is in the up state (13,14).

Movement of the transmembrane (TM) helices therefore appears to regulate channel activity, but unlike many other K^+ channels, this movement does not appear to constrict the pore to switch it between an open and closed state because TREK channels are gated primarily within their selectivity filter (11,15,16). The mechanism by which membrane tension shifts the channel into the more active up state has been studied extensively (4,17,18). However, the mechanisms by which regulatory lipids such as PIP_2 interact with TREK channels to influence these conformations remains unclear.

In this study, we have used both coarse-grained and atomistic molecular dynamics (MD) simulations to model PIP_2 binding to different conformations of TREK-1 and calculate its energetics of binding relative to other anionic phospholipids. Our results indicate that PIP_2 promotes a conformational transition toward the up state and proposes a mechanism of activation by PIP_2 that is consistent with a wide range of evidence from previous binding studies and functional assays and with mutagenesis data.

MATERIALS AND METHODS

Coarse-grained (CG) simulation set up

The TREK-1 amino acid sequence was taken from UniProt: O95069. The sequence was then aligned to TREK-2 structure (PDB: 6CQ6 – TREK-1 up state, (11); PDB: 4XDJ – TREK-2 down state (10)) using SWISS-MODEL to generate two all-atom structures (19). We generated a two-fold symmetry structure for each conformational state of TREK-1 by transplanting the structure of one subunit onto another using PyMOL (20). As the down state does not have an extended C-terminus structure, we extended this segment of TREK-1 by superimposing the α-helical structure based on the subunit A from 6CQ6. The structure was converted from atomistic to coarse grained using martinize.py (21). In this study, all CG simulations were calculated using the Martini2.3 force field. The protein is then placed in the periodic simulation box at a minimum distance of 15 Å from the box edge in all directions.

Palmitoyl-2-oleoylphosphatidylcholine (POPC) is a common lipid headgroup in a mammalian plasma membrane (22). We use palmitoyl oleyl as the lipid tail for all lipid headgroups (PIP_2, phosphatidylinositol-4-phosphate (PI4P), phosphatidylinositol (PI), phosphatidylglycerol (PG), phosphatidic acid (PA), phosphatidylserine (PS)). Bilayers were assembled around the TREK-1 and flooded with the CG water particles using insane.py, with a similar area per lipid between both the upper and the lower leaflet (23). The bilayers have ratios of POPC:POPS:PIP_2 at 1:0:0 for the upper leaflet and 15:3:2 for the lower leaflet. We added POPS as PS is localized exclusively in the cytoplasmic leaflet and accounts for 13%–15% of anionic phospholipids in the human cerebral cortex. The CG model of TREK-1 was aligned perpendicular to the bilayer. 0.15 mM NaCl was then generated using genion to replace water particles with sodium (Na^+) and chloride (Cl^-) ions. Together, each simulation box has a size of $13 \times 13 \times 13$ nm^3, containing ~19,000 CG beads (24).

CG-MD simulations

Simulations were run with GROMACS 2020.1 using the V-rescale thermostat for temperature coupling (25) and the Parrinello-Rahman barostat for semi-isotropic pressure (26). The temperature was coupled to 310 K, and the pressure was kept at 1 bar in the *xy* direction. We applied a positional restraint force of 1000 kJ mol^{-1} nm^{-2} to the backbone beads to maintain their structure. Energy minimization was carried out using the steepest descent algorithm with an energy cap of 1000 kJ mol^{-1} nm^{-2}. The system was then equilibrated for 100 ps with the Berendsen thermostat algorithm (27). For lipid-binding site analysis, each production MD was performed for 10 μs with 20 fs timesteps. The binding sites were then assessed using pylipid.py (28). The interactions were counted when the distance between amino-acid residue and lipid headgroup was shorter than 0.6 nm, and we stopped counting when the distance was further than 0.8 nm.

CG-PMF calculations

Snapshots of the final frame were taken from CG-MD simulations for the potential of mean force (PMF) calculation. TREK-1 and a single PIP_2 in the target binding site were isolated using PyMOL (20) and re-assembled into POPC bilayers using insane.py (23). The system was energy minimized and equilibrated with PIP_2, and the protein backbone was restrained for 15 ns. The system was then equilibrated further without PIP_2 restrained for an additional 15 ns. All position restraints were carried out at 1000 kJ mol^{-1} nm^{-2}. To maintain a structural consistency with the crystal structures, the protein was restrained throughout the PMF calculation. PIP_2 was then pulled from the binding site using steered MD along the collective variable (CV). We defined the CV as the distances between the PIP_2 headgroup and reference points. We defined our reference points as the backbone particle of the residue near the PIP_2-binding site (D294 for the up state, R297 for the down state). To pull the headgroup, we applied an elastic force (1000 kJ mol^{-1} nm^{-2}) to pull the PIP_2 headgroup along the CV as described. During our umbrella sampling, we sampled CVs with 0.5 Å spacing for optimal histogram overlap. These snapshots are used to initialize independent MD simulations where umbrella potential with the force of 1000 kJ mol^{-1} nm^{-2} was applied to the PIP_2 headgroup in each window. MD simulations were performed for 1.2 μs to allow for convergence, and the first 200 ns was removed from each window as equilibration. These windows were then used to generate 1D energy landscapes using the weighted histogram analysis method from the gmx wham tool with 200 Bayesian bootstraps (29).

CG-FEP

The bound positions of PIP_2 were taken from the PMF simulations and used as input for the free-energy perturbation (FEP) calculations. The PIP_2 headgroup was alchemically transformed into other lipid headgroups (PI4P, PI, PG, PS, PA) using an approach similar to our previous study (30,31). This transformation was done in a chemical space with λ as a coordinate parameter. All transformations were performed separately in both bound states and in bulk bilayer to create complete thermodynamic cycles.

During transformations, CG beads were altered to change PIP_2 into other lipids. Some beads were transformed into dummies with no interaction properties or different beads with properties matching the target. These alteration choices were made manually by aligning PIP_2 with other lipids structures. Each transformation was performed in steps where λ was slowly changed from 0 (PIP_2) to 1 (other lipids). As in this article, each transformation was split into 15 windows where Coulombic and van der Waals interactions were transformed separately, with soft-core parameters ($\alpha = 0.5$ and $\sigma = 0.3$) for both interactions, similar to our previous study (32). Coulombic interactions' λ parameter was perturbed linearly in the first nine windows and converged to one at the end (λ = 0.00, 0.10, 0.20, …, 1.00), while van der Waals interactions' λ parameter was perturbed linearly started from the sixth window toward the final window (λ = 0.00 … 0.00, 0.10, 0.20, …, 0.90, 1.00). Energy minimizations were performed with the steepest descent algorithm for 200 steps. Fifteen independent production simulations with randomized initial velocities were then run using leap-frog stochastic dynamics integrator to 12 ns per window, where the first 2 ns was discarded as equilibration. Using the alchemical-analysis software package, we constructed free-energy pathways from individual simulations window with Multistate Bennett acceptance ratio (33).

Atomistic simulations

The CG-MD simulation snapshots were taken to isolate the channel with PIP_2 in the most prominent binding site. We generated a position of PIP_2 by transplanting the position of the PIP_2 in the binding site onto another subunit using PyMOL. We then re-assembled the PIP_2-bound TREK-1 into the POPC bilayer using insane.py. Energy minimization and equilibration were carried out using the same method used in CG-PMF. The original CG structures of up and down state TREK-1 were inserted into a 100% POPC bilayer using insane.py for an unbound TREK-1 simulation. The system was energy minimized using the steepest-descent algorithm and equilibrated with protein backbone restrained for 30 ns at 1000 kJ mol^{-1} nm^{-2} using V-rescale thermostat for temperature coupling (25) and the Berendsen barostat for semi-isotropic pressure coupling (27).

The up and down state CG structures were converted to atomistic structures using cg2at.py (34). We inserted potassium (K^+) ions from the crystal structures into the selectivity filter and converted Na^+ ions into K^+ ions. Thus, our system in atomistic simulation is solvated in a 0.15 KCl solution. Another energy minimization was performed to adjust the position of the K^+ ions inside the selectivity filter. The system was then energy minimized using steepest-descent algorithm for 5000 steps and then equilibrated for 10 ns with the restraint of 1000 kJ nm^{-2} mol^{-1} on the C_α atoms. During the equilibration, all systems (both with and without PIP_2) were coupled with V-rescale thermostat to maintain simulation temperature at 310 K (25) and the Berendsen barostat for semi-isotropic pressure coupling (27). Three 500 ns production simulations for each state were performed with different randomized initial velocities. During the production run, V-rescale thermostat was used to maintain the simulation temperature at 310 K (25) and the Parrinello-Rahman barostat for semi-isotropic pressure at 1 bar in the *xy* direction (26). All atomistic simulations and analyses (root-mean-square fluctuation, hydrogen bonding, principal-component analysis, and distance calculation) were performed using GROMACS 2020.1 with CHARMM36 force fields (24,35,36). 95% confidence interval from root-mean-square fluctuation (RMSF) analysis from each simulation was calculated using $CI_{\Delta RMSF} = \sqrt{CI_{RMSF_{PIP_2}} + CI_{RMSF_{APO}}}$. We assessed the amino-acid residues that interact with the anionic headgroup of the PIP_2 using pylipid.py with the cut-off distance of 0.4 nm.

RESULTS

Identification of a PIP_2-binding site

We conducted 10 μs CG-MD simulations to investigate putative lipid-binding sites in the up-state (PDB: 6CQ6) (11) and the down-state (PDB: 4XDJ) (10) conformations of TREK-1. The distal C-terminus of the down-state structure was extended to match the longer α-helical structure that was resolved in the up-state structure of TREK-1 (PDB: 6CQ6). In our simulations, PIP_2 was initially placed randomly in the lower leaflet with the mixture of PC:PS:PIP_2 at the ratio of 15:3:2 and simulated for 10 μs for each structure (Fig. 1 *A*). Similar to previous protein-PIP_2 interaction studies (30,37,38), we used two criteria to identify possible PIP_2-binding sites. First, the "residence time" of the PIP_2 in its binding site was assessed, i.e., the period in which PIP_2 remained at least 0.6 nm from the residues of interest. Second, we assessed the "occupancy," defined as the fraction of time that PIP_2 spends at least 0.6 nm in proximity to a particular residue. These metrics allowed us to cluster any residues with similar residence time and occupancy, therefore defining them as a potential interaction or binding sites.

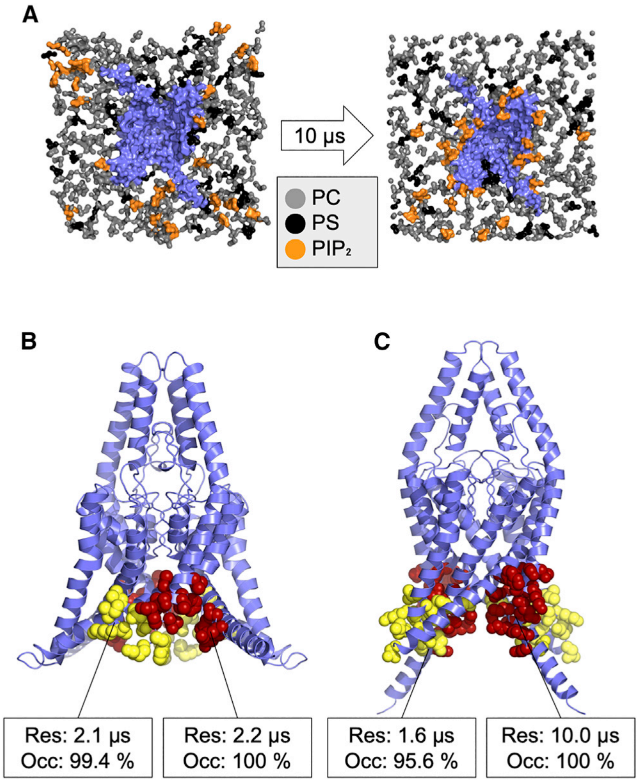

FIGURE 1 Coarse-grained molecular dynamics simulation. (*A*) Schematic representation of the coarse-grained simulation conducted in this study. Only the headgroup of the lower leaflet lipids (PC: *gray*, PS: *black*, and PIP_2: *orange*) are shown. TREK-1 (*blue*) in the up state is embedded in the phospholipid bilayer. After a 10 μs simulation (*right*), PIP_2 headgroups are distributed more closely at their relative binding site (*right*) compared with their initial distribution (*left*). (*B* and *C*) The PIP_2-binding site on the TREK-1 (*B*) up state or (*C*) down state. Binding sites 1 (*red*) and 2 (*yellow*) are shown superimposed relative to their crystal structure. Each site is annotated with their residence time within the binding site and their occupancy. To see this figure in color, go online.

We identified two binding sites with occupancy >90% and residence times >2 μs (Figs. 1 *B* and *C*, S1, and S2). Other sites with residence times less than 0.5 μs were treated as non-specific interactions. In the up state, the two binding sites were adjacent to each other and located between M1 and M4 (Fig. 1 *B*). In contrast, the binding sites on the down-state structure were further away from each other (Fig. 1 *C*), and the binding site with the highest residence time was similar to the site identified in the up state. However, the second binding site in this conformation was located at the M2/M3 interface, and the two binding sites were separated from each other by the M2 helix. In contrast, these two sites were much closer together when the channel was in the up state (Fig. 1 *B*).

PIP_2-binding affinities between up and down states

Similar to a previous approach (31), we next assessed the binding affinity of PIP_2 in the most prominent binding site using PMF calculations to calculate the free energy of binding. We compared the energetic difference between the "bound" state (energy minima) and the "bulk," where lipids are at least 1.2 nm away from the binding site (Fig. 2 *A*). We showed that the binding free energy in the most occupied binding sites are -41 ± 3 and -26 ± 2 kJ mol^{-1} for the up and down states, respectively (Fig. 2 *B*). Thus, the binding of PIP_2 appears more favorable in the up state than in the down state. We also observed a shallower energy landscape in the down state, implying less specificity in the binding site.

Comparing lipid specificity in the primary binding site

To determine the relative binding free energy between PIP_2 and other lipids in this binding site, we applied the FEP method to perturb the PIP_2 headgroup to other lipid headgroups. Relative binding free energies were then calculated relative to phosphatidylcholine (PC) (Fig. 2 *C* and *D*). We define the binding free energy of PC to TREK-1 channel to be ~0 kJ mol^{-1}, as this is the most common lipid in the bilayer. In the up state, the binding free energy difference between PIP_2 and PC is -36 ± 2 kJ mol^{-1}, which agrees well with our PMF calculations (-41 ± 3 kJ mol^{-1}). This value is also remarkably close to the value calculated in a fluorescent binding assay that yielded -36 ± 1 kJ mol^{-1} from a dissociation constant of 0.80 ± 0.34 μM (39).

By comparing these binding free energies relative to PC, we observed that channel affinity for PIP_2 is slightly higher than for PI4P. This suggests that the first phosphate group contributes only partially to the binding of PIP_2. However, the transformation from PIP_2 to PI showed that the inositol group and the second phosphate group have a much more significant contribution than the first phosphate group. This pattern has also been observed in inwardly rectifying K^+ channels (30). In addition, we compared the relative binding free energy of PIP_2 wtih other anionic phospholipids such as PG, PS, and phosphatidic acid (PA) (Figs. 2 *D* and S3). This showed that the binding free energy of PG, PS, and PA are relatively similar (-10 kJ mol^{-1} relative to PC).

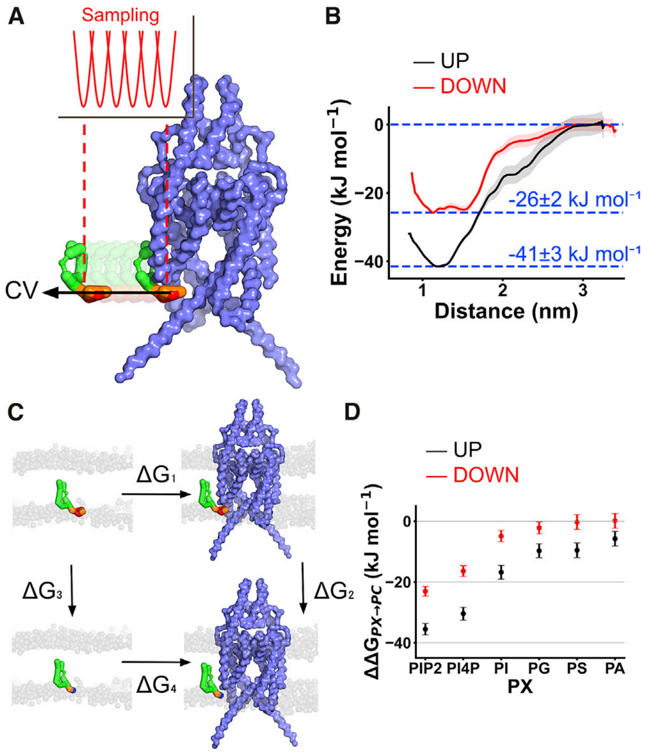

FIGURE 2 Calculation of PIP_2-binding free energy relative to other lipids. (*A*) Schematic description of the coarse-grained potential of mean force (CG-PMF) calculations. In this scheme, PIP_2 (*green*) in the binding site was steered away from the protein (*blue surface*) along the collective variable (*arrow*). The PMF was calculated using umbrella sampling with the force constraint at 1000 kJ $nm^{-2}mol^{-1}$ (*red*). (*B*) One-dimensional energy landscape calculated from PMF calculation as PIP_2 is steered away from the binding site from up (*black*) or down (*red*) states to the bulk lipid bilayer (PC). Energy landscape is marked at 0 kJ mol^{-1} when the PIP_2 is >1.2 nm away from the binding site. (*C*) Schematic description of CG free-energy perturbation. The PIP_2 headgroup was alchemically transformed to different lipids (PI4P, PC, PS, PG, PA) in both the bound state (ΔG_2) and the bulk state (ΔG_3). This transformation allows relative affinities between two lipids species to be calculated. (*D*) Binding free energy of PIP_2, PI4P, PI, PG, PS, and PA relative to PC within the TREK-1 PIP_2-binding site in the up (*black*) or down state (*red*). Error bars showed 95% confidence interval calculated based on t-statistics (n = 15). To see this figure in color, go online.

We next conducted a similar CG-FEP calculation using the structure of the down-state TREK-1. Here, we observed a similar difference between the affinity of PIP_2, PI4P, and PI compared with the up state. However, we also observed a much smaller binding energy of PG, PS, and PA (~0 kJ mol^{-1}relative to PC) in the down state, suggesting that any channel activation of these anionic phospholipids is only likely to occur in the up state.

A previous study suggested that PA activates the channel because it competes PIP_2 away from the binding site. However, our free-energy calculation showed that the PA affinity is much lower than PIP_2 in the up state. Thus, we examined whether PA may accommodate an alternative binding site to the channel or possibly accumulate near the PIP_2-binding site. To do so, we prepared a bilayer containing 10% PA and 90% PC and conducted a 10 μs unbiased simulation to identify PA binding to the channel. In the up state, the site where PA binds with the highest occupancy and the longest residence time is not the same site as the PIP_2-binding site. This PA-binding site is located between M1 and M2 helices, whereas the PIP_2 site is located between M1 and M4 helices. At the PIP_2 site, the occupancy and residence time of PA are much lower than for PIP_2 (0.46 μs in PA-binding site and 2.21 μs in PIP_2-binding site) (Fig. S4). This suggests that PA is unlikely to directly compete with PIP_2 at the PIP_2-binding site we have identified.

Atomistic simulation of TREK-1

Full atomistic simulations allow us to observe conformational changes in the M4 helix and can capture the geometry of the binding pocket at a much higher resolution. We therefore took snapshots from the final frame of the CG-MD simulations and used these to seed atomistic simulations. To assess the geometry of the binding pocket, we ran 500 ns simulations of both up and down states with a single PIP_2 molecule in the binding site in a bulk PC bilayer ($n = 3$). As TREK-1 is a homodimer, the data collected from each subunit were treated as a single data point ($n = 6$). We defined contact residues as those that spent more than 50% of the simulation time at < 4 Å proximity of the PIP_2 headgroup.

In the up state, PIP_2 coordinates with 3 residues on M4 (R297, K301, and K304). These three residues are all on the same helical interface of M4 (Fig. 3 *A*). Within this binding site, R297 and K304 are coordinated between two phosphate groups (P1 and P5) on the inositol ring (Figs. 3 *A* and S5), whereas K301 was only coordinated to P5. However, the P4 phosphate group was not coordinated by any amino-acid residues. These variations may explain the lower relative binding free-energy differences when the first phosphate group was perturbed in our CG-FEP calculation.

In the down state, PIP_2 only coordinates with 2 residues (K304 and R311). Both are on M4 and form a site that is further away from the pore domain than the binding site in the up state (Fig. 3 *B*). The phosphate group connected to C4 is also singly coordinated by R311, whereas C5 is coordinated by K304. Interestingly, the C1 phosphate is not coordinated by any amino acid residues within the C-terminus. The fewer contacts made in the down state may therefore explain the lower binding free energy obtained from the PMF calculation.

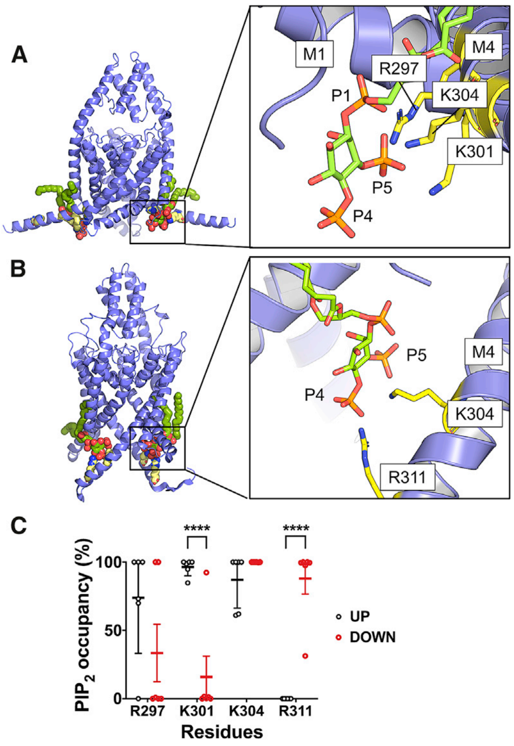

FIGURE 3 Atomistic simulation of PIP_2 and TREK-1. (*A* and *B*) Representative interactions between (*A*) up state and (*B*) down state of TREK-1. The backbone of the protein is represented in blue. Key contacting residues contacting to the phosphate group on the PIP_2 molecule are shown in yellow. (*C*) PIP_2-contact analysis showing the fraction of time that residues are in 4 Å proximity to the PIP_2 molecule (contact probability) of the up state (*black*) and down state (*red*) of TREK-1. Only residues with an average >50% contact probability are shown. Data are collected from three repeats of 500 ns simulations. Error bars showed 95% confidence interval calculated based on t-statistics ($n = 6$). Structural models highlighting these binding sites are also available (Supplementary Data S1 and S2). To see this figure in color, go online.

Comparison of the coordination pattern between the up and down states revealed that PIP_2 was in contact with K301 more often in the up state than the down state ($n = 6$, Student's *t*-test: $p < 0.0001$). On the other hand, coordination with R311 was significantly more favorable in the down state ($n = 6$, Student's *t*-test: $p < 0.0001$) (Fig. 3 *C*). We then calculated an average number of hydrogen bonds formed in the last 100 ns of the simulation (Fig. S5). Assuming that the interaction was driven mostly by electrostatic interaction, this suggests that the up state exhibits a greater degree of hydrogen bonding with the key PIP_2-interacting residues than the down state. This therefore allows us to postulate that PIP_2 binding is stabilized in the up state but not in the down state.

Effect of PIP_2 on dynamics of the C-terminus

We next investigated the conformational dynamics of TREK-1 in three 500 ns atomistic simulations of both PIP_2-bound and -unbound states. To assess the protein

backbone dynamics, we calculated the RMSF on the Cα atom on the protein and compared the effect of PIP_2 relative to the unbound state (control). This difference (ΔRMSF) is shown in Fig. 4 *A*. This shows that M4 and the C-terminus fluctuates more when PIP_2 is absent in the down state (ΔRMSF ~8 Å) and suggests that PIP_2 may stabilize the conformation of the C-terminus in the down state. However, the dynamics of the C-terminus are relatively unaffected by PIP_2 in the up state, as the PC headgroups may stabilize the α-helical content of the M4 helix (Fig. 4 *A*). The reason that the C-terminus of the up state is likely to be more stable than the down-state is because this region appears to interact with the phosphate headgroups of the phospholipids when closer to the membrane in the up state. On the other hand, none of the TREK-1 down state crystal structures are solved with this part of the C-terminus resolved. Our simulations strongly suggest that this region is highly flexible and dynamic in the down state but may become stabilized upon PIP_2 binding.

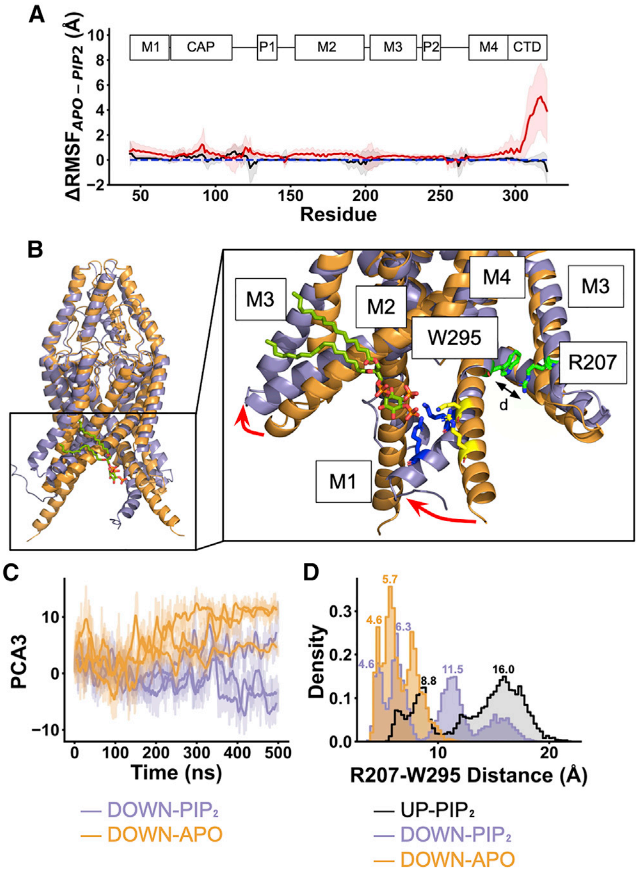

FIGURE 4 PIP_2 induces conformational change on TREK-1 C-terminus. (*A*) The difference between the C_α RMSF in the presence and absence of PIP_2 (ΔRMSF) of the up state (*black*) and down state (*red*) of TREK-1. The positive ΔRMSF value implies that the backbone is stabilized by PIP_2. Shaded region represents the propagation of 95% confidence interval. (*B*) The conformational change induced by PIP_2 in the down state. The initial conformation at 0 ns (*orange*) is compared with the post-simulation at 500 ns (*blue*) with PIP_2 (*dark green*) in the binding site. The key conformational change in the C-terminal domain is described with the red arrow. We defined the zipper measurement based on the distance between R207 and W295. Residues coordinated with PIP_2 (K304 and R311) are highlighted before (*yellow*) and after (*blue*) 500 ns simulation. (*C*) The magnitude of the conformational change along the vector describing the third principal component from the down state plotted against time in the unbound state (*orange*) and PIP_2-bound state (*purple*). The darker lines show the running average for each simulation (500 ns) (n = 3). (*D*) Distribution plot of the zipper distance collected from the final 100 ns of each simulation for the PIP_2-bound up state (*black*), the PIP_2-bound down state (*purple*), and the unbound down state (*orange*). To see this figure in color, go online.

PIP_2-induced conformational transition

We then focused on the down state, where PIP_2 had large effects on the dynamics of the C-terminus. Over 500 ns, our contact analysis showed that K304 and R311 on M4 bend to interact more closely with PIP_2 (Fig. 3 *B* and *C*). This bending enables M2/M3 to move upward. Together, this widens the cavity between M2 and M3 (Fig. 4 *B*). To quantify the functional dynamics of TREK-1 when PIP_2 is bound, we calculated the first and the second eigenvectors on the protein backbone motion using principal-component analysis. This decomposed the backbone movement relative to the initial structure into orthonormal bases (components). We then projected the movements from the simulations on these components to observe the principal movement ordered by their eigenvalues. The first and second principal components capture the motion of the extracellular cap domain (Fig. S6) and we focused on the third principal component (PC3), where the conformational change at the C-terminus occurs (Fig. 4 *C*).

To quantify this change in PC3 (7%), we used the "zipper" measurement between W295 in M4 and R207 in M3 on the same chain (4) and plotted the distribution of this distance (Figs. 4 *D* and S7). When compared with the up state, where no conformational change occurred at the C-terminus in response to PIP_2, the transition from the down to the up state involved a re-orientation of W295 in the lower part of M4. This transition breaks its interaction with R207, which then allows an expansion between TM2 and TM4. We therefore used the distance between the center of mass of R207 and W295 as a metric of the down-to-up transition (Figs. 4 *D* and S7) and observed a bimodal distribution, where the majority of the population is distributed between 10 and 20 Å and a minority at ~8 Å.

In contrast, the distance between R207 and W295 was only distributed around 3–9 Å in the down state in the absence of PIP_2. The down state also displayed a unique population at about 11.5 Å, similar to the up state (Fig. 4 *D*). This suggests that this simulation may capture an initial path of a down-to-up conformational transition that is driven by interaction with PIP_2.

DISCUSSION

This study used CG-MD simulation to predict PIP_2-binding sites on TREK-1 and highlighted differences in PIP_2 binding

between up and down states of the channel. We also quantified the binding free energy of different lipids in both conformations and used principal-component analysis to examine the conformational changes induced by PIP_2. Our results not only identify key residues involved in PIP_2 binding but also show that PIP_2 induces a conformational transition from the down to the up state.

Although PIP_2 has a clear activatory effect on TREK channels (5,7,40–43), inhibitory effects of PIP_2 have also been reported (44–46). In addition to PIP_2, other anionic phospholipids such as PA have also been shown to increase channel activity (7) and in one particular study (46), PIP_2 was shown to have a concentration-dependent biphasic effect on the channel, being activatory at low concentration and inhibitory at high concentration. The activatory effect is proposed to result from PIP_2 being hydrolyzed to PA by phospholipase D2 bound to the C-terminus of TREK channels, with the increase in local concentration of PA directly activating TREK-1 (8); by contrast, the inhibitory effect is proposed to result from PIP_2 competing PA out of its activatory site, implying that PA may be the main channel activator, not PIP_2 (39).

However, our results are more consistent with direct activation by interaction with PIP_2 and, importantly, are consistent with mutagenesis and other functional studies as well as with biochemical studies of lipid binding. The putative PIP_2-binding site we identify in the up state includes residues R297, K301, and K304, which have previously been implicated in PIP_2 binding (7). Also, the PIP_2-binding site we identify in the down state involves K304 and R311, and mutation of R311 also affects channel activity in a way that is consistent with its role in PIP_2 binding (7). Our results therefore support the idea that PIP_2 directly interacts with this region of the proximal C-terminus (7). Furthermore, our simulation proposes that PIP_2 binding to the channel changes as it transits from the down to the up state.

We also show that PIP_2 has a lower binding affinity to the down state than to the up state, and our atomistic simulations indicate that in the presence of PIP_2, the down state begins to transit toward the up state. This model is consistent with other studies (12,13), and computational electrophysiology studies also suggest that the down state of TREK-2 is less active than the up state (14); similar results have recently been suggested for TREK-1 (47).

This is also supported by single-channel studies of the related TRAAK channel that suggest that the up state is more conductive (48). Thus, we propose that PIP_2 activates TREK-1 by promoting a transition from the down state to an up state that is capable of supporting a higher degree of channel activity. This change involves an expansion of the distance between M2 and M4 and is accompanied by changing the coordination of PIP_2 from R311 to K304 in the PIP_2-binding site.

We have also assessed the affinity of different anionic phospholipids in the PIP_2-binding site of TREK-1. Overall, the binding affinity of anionic phospholipids is much lower in the down state than in the up state. Our simulations also shows that PA affinity to the channel is approximately fourfold lower than for PIP_2 in the up state, whereas this affinity is almost negligible in the down state. This suggests that PA may only bind and activate the channel when it is in the up state with PIP_2 already bound to the channel, though the mechanism involved remains unclear.

Overall, these simulations provide additional mechanistic insight and understanding of the conformational dynamics of TREK-1 and its activation by PIP_2 as well as by other anionic phospholipids. In addition, they provide a better understanding of the different mechanisms of state-dependent lipid activation of TREK channels and the complex allosteric processes involved in their polymodal activation by different signaling pathways.

SUPPORTING MATERIAL

Supporting material can be found online at https://doi.org/10.1016/j.bpj.2022.05.019.

AUTHOR CONTRIBUTIONS

A.P. conducted all MD simulations. All authors jointly designed the research, analyzed the data, and wrote the manuscript. S.J.T. acquired the funding.

ACKNOWLEDGMENTS

This work was supported by grants from the Biotechnological and Biological Sciences Research Council to S.J.T and by an OXION PhD Program Studentship from the Wellcome Trust, United Kingdom to T.P. A.P. was funded by a Royal Thai Government Scholarship. We thank Viwan Jarerattanachat for fruitful discussions and inspiration. We also thank members of the Tucker, Sansom, Stansfeld, and Biggin groups for their helpful comments during the development of this project.

DECLARATION OF INTERESTS

The authors declare no conflicts of interest in this study.

REFERENCES

1. Natale, A. M., P. E. Deal, and D. L. Minor, Jr. 2021. Structural insights into the mechanisms and pharmacology of K2P potassium channels. *J. Mol. Biol.* 433:166995. https://doi.org/10.1016/j.jmb.2021.166995.
2. Enyedi, P., and G. Czirják. 2010. Molecular background of leak K+ currents: two-pore domain potassium channels. *Physiol. Rev.* 90:559–605. https://doi.org/10.1152/physrev.00029.2009.
3. Busserolles, J., I. Ben Soussia, ..., S. Lolignier. 2020. TREK1 channel activation as a new analgesic strategy devoid of opioid adverse effects. *Br. J. Pharmacol.* 177:4782–4795. https://doi.org/10.1111/bph.15243.
4. Aryal, P., V. Jarerattanachat, ..., S. J. Tucker. 2017. Bilayer-mediated structural transitions control mechanosensitivity of the TREK-2 K2P channel. *Structure.* 25:708–718.e2. https://doi.org/10.1016/j.str.2017.03.006.
5. Riel, E. B., B. C. Jürs, ..., T. Baukrowitz. 2022. The versatile regulation of K2P channels by polyanionic lipids of the phosphoinositide and fatty

acid metabolism. *J. Gen. Physiol.* 154. . e202112989. https://doi.org/10.1085/jgp.202112989.

6. Maingret, F., A. J. Patel, …, E. Honoré. 1999. Mechano- or acid stimulation, two interactive modes of activation of the TREK-1 potassium channel. *J. Biol. Chem.* 274:26691–26696. https://doi.org/10.1074/jbc.274.38.26691.
7. Chemin, J., A. J. Patel, …, E. Honoré. 2005. A phospholipid sensor controls mechanogating of the K+ channel TREK-1. *EMBO J.* 24:44–53. https://doi.org/10.1038/sj.emboj.7600494.
8. Comoglio, Y., J. Levitz, …, G. Sandoz. 2014. Phospholipase D2 specifically regulates TREK potassium channels via direct interaction and local production of phosphatidic acid. *Proc. Natl. Acad. Sci. U S A.* 111:13547–13552. https://doi.org/10.1073/pnas.1407160111.
9. Petersen, E. N., M. Gudheti, …, S. B. Hansen. 2019. Phospholipase D transduces force to TREK-1 channels in a biological membrane. Preprint at bioRxiv. https://doi.org/10.1101/758896.
10. Dong, Y. Y., A. C. W. Pike, …, E. P. Carpenter. 2015. K2P channel gating mechanisms revealed by structures of TREK-2 and a complex with Prozac. *Science.* 347:1256–1259. https://doi.org/10.1126/science.1261512.
11. Lolicato, M., C. Arrigoni, …, D. L. Minor. 2017. K2P2.1 (TREK-1)–activator complexes reveal a cryptic selectivity filter binding site. *Nature.* 547:364–368. https://doi.org/10.1038/nature22988.
12. McClenaghan, C., M. Schewe, …, S. J. Tucker. 2016. Polymodal activation of the TREK-2 K2P channel produces structurally distinct open states. *J. Gen. Physiol.* 147:497–505. https://doi.org/10.1085/jgp.201611601.
13. Proks, P., M. Schewe, …, S. J. Tucker. 2021. Norfluoxetine inhibits TREK-2 K2P channels by multiple mechanisms including state-independent effects on the selectivity filter gate. *J. Gen. Physiol.* 153:e202012812. https://doi.org/10.1085/jgp.202012812.
14. Brennecke, J. T., and B. L. de Groot. 2018. Mechanism of mechanosensitive gating of the TREK-2 potassium channel. *Biophys. J.* 114:1336–1343. https://doi.org/10.1016/j.bpj.2018.01.030.
15. Piechotta, P. L., M. Rapedius, …, T. Baukrowitz. 2011. The pore structure and gating mechanism of K2P channels. *EMBO J.* 30:3607–3619. https://doi.org/10.1038/emboj.2011.268.
16. Bagriantsev, S. N., R. Peyronnet, …, D. L. Minor. 2011. Multiple modalities converge on a common gate to control K2P channel function. *EMBO J.* 30:3594–3606. https://doi.org/10.1038/emboj.2011.230.
17. Clausen, M. V., V. Jarerattanachat, …, S. J. Tucker. 2017. Asymmetric mechanosensitivity in a eukaryotic ion channel. *Proc. Natl. Acad. Sci. U S A.* 114:E8343–E8351. https://doi.org/10.1073/pnas.1708990114.
18. Rietmeijer, R. A., B. Sorum, …, S. G. Brohawn. 2021. Physical basis for distinct basal and mechanically gated activity of the human K(+) channel TRAAK. *Neuron.* 109:2902–2913.e4. https://doi.org/10.1016/j.neuron.2021.07.009.
19. Waterhouse, A., M. Bertoni, …, T. Schwede. 2018. SWISS-MODEL: homology modelling of protein structures and complexes. *Nucleic Acids Res.* 46:W296–W303. https://doi.org/10.1093/nar/gky427.
20. Schrodinger LLC. 2015. . The PyMOL Molecular Graphics System, Version 1.8. Schrodinger LLC.
21. Monticelli, L., S. K. Kandasamy, …, S.-J. Marrink. 2008. The MARTINI coarse-grained force field: extension to proteins. *J. Chem. Theory Comput.* 4:819–834. https://doi.org/10.1021/ct700324x.
22. Casares, D., P. V Escribá, and C. A. Rosselló. 2019. Membrane lipid composition: effect on membrane and organelle structure, function and compartmentalization and therapeutic avenues. *Int. J. Mol. Sci.* 20:2167. https://doi.org/10.3390/ijms20092167.
23. Wassenaar, T. A., H. I. Ingólfsson, …, S. J. Marrink. 2015. Computational lipidomics with insane: a versatile tool for generating custom membranes for molecular simulations. *J. Chem. Theory Comput.* 11:2144–2155. https://doi.org/10.1021/acs.jctc.5b00209.
24. Abraham, M. J., T. Murtola, …, E. Lindahl. 2015. Gromacs: high performance molecular simulations through multi-level parallelism from laptops to supercomputers. *SoftwareX.* 1–2:19–25. https://doi.org/10.1016/j.softx.2015.06.001.
25. Bussi, G., D. Donadio, and M. Parrinello. 2007. Canonical sampling through velocity rescaling. *J. Chem. Phys.* 126:014101. https://doi.org/10.1063/1.2408420.
26. Parrinello, M., and A. Rahman. 1981. Polymorphic transitions in single crystals: a new molecular dynamics method. *J. Appl. Phys.* 52:7182–7190. https://doi.org/10.1063/1.328693.
27. Berendsen, H. J. C., J. P. M. Postma, …, J. R. Haak. 1984. Molecular dynamics with coupling to an external bath. *J. Chem. Phys.* 81:3684–3690. https://doi.org/10.1063/1.448118.
28. Song, W., R. A. Corey, …, M. S. P. Sansom. 2021. PyLipID: a Python package for analysis of protein-lipid interactions from MD simulations. Preprint at bioRxiv. https://doi.org/10.1101/2021.07.14.452312.
29. Hub, J. S., B. L. De Groot, and D. Van Der Spoel. 2010. g_wham-A free weighted histogram analysis implementation including robust error and autocorrelation estimates. *J. Chem. Theory Comput.* 6:3713–3720. https://doi.org/10.1021/ct100494z.
30. Pipatpolkai, T., R. A. Corey, …, P. J. Stansfeld. 2020. Evaluating inositol phospholipid interactions with inward rectifier potassium channels and characterising their role in disease. *Commun. Chem.* 3:147. https://doi.org/10.1038/s42004-020-00391-0.
31. Corey, R. A., O. N. Vickery, …, P. J. Stansfeld. 2019. Insights into membrane protein-lipid interactions from free energy calculations. *J. Chem. Theory Comput.* 15:5727–5736. https://doi.org/10.1021/acs.jctc.9b00548.
32. Corey, R. A., W. Song, …, P. J. Stansfeld. 2021. Identification and assessment of cardiolipin interactions with E. coli inner membrane proteins. Preprint at bioRxiv. https://doi.org/10.1101/2021.03.19.436130.
33. Fajer, M., R. V Swift, and J. A. McCammon. 2009. Using multistate free energy techniques to improve the efficiency of replica exchange accelerated molecular dynamics. *J. Comput. Chem.* 30:1719–1725. https://doi.org/10.1002/jcc.21285.
34. Vickery, O. N., and P. J. Stansfeld. 2021. CG2AT2: an enhanced fragment-based approach for serial multi-scale molecular dynamics simulations. Preprint at bioRxiv. https://doi.org/10.1101/2021.03.25.437005.
35. Huang, J., and A. D. Mackerell. 2013. CHARMM36 all-atom additive protein force field: validation based on comparison to NMR data. *J. Comput. Chem.* 34:2135–2145. https://doi.org/10.1002/jcc.23354.
36. Huang, J., S. Rauscher, …, A. D. MacKerell. 2017. CHARMM36m: an improved force field for folded and intrinsically disordered proteins. *Nat. Methods.* 14:71–73. https://doi.org/10.1038/nmeth.4067.
37. Stansfeld, P. J., R. Hopkinson, …, M. S. P. Sansom. 2009. PIP2-binding site in Kir channels: definition by multiscale biomolecular simulations. *Biochemistry.* 48:10926–10933. https://doi.org/10.1021/bi9013193.
38. Duncan, A. L., R. A. Corey, and M. S. P. Sansom. 2020. Defining how multiple lipid species interact with inward rectifier potassium (Kir2) channels. *Proc. Natl. Acad. Sci. U S A.* 117:7803–7813. https://doi.org/10.1073/pnas.1918387117.
39. Cabanos, C., M. Wang, …, S. B. Hansen. 2017. A soluble fluorescent binding assay reveals PIP2 antagonism of TREK-1 channels. *Cell Rep.* 20:1287–1294. https://doi.org/10.1016/j.celrep.2017.07.034.
40. Chemin, J., A. Patel, …, E. Honoré. 2005. Lysophosphatidic acid-operated K+ channels. *J. Biol. Chem.* 280:4415–4421. https://doi.org/10.1074/jbc.m408246200.
41. Lopes, C. M. B., T. Rohács, …, D. E. Logothetis. 2005. PIP2 hydrolysis underlies agonist-induced inhibition and regulates voltage gating of two-pore domain K+ channels. *J. Physiol.* 564:117–129. https://doi.org/10.1113/jphysiol.2004.081935.
42. Soussia, I. B., F. S. Choveau, …, F. Lesage. 2018. Antagonistic effect of a cytoplasmic domain on the basal activity of polymodal potassium channels. *Front. Mol. Neurosci.* 11:301. https://doi.org/10.3389/fnmol.2018.00301.

43. Rivas-Ramírez, P., A. Reboreda, …, J. A. Lamas. 2020. PIP2 mediated inhibition of TREK potassium currents by bradykinin in mouse sympathetic Neurons. *Int. J. Mol. Sci.* 21:389. https://doi.org/10.3390/ijms21020389.

44. Chemin, J., A. J. Patel, …, E. Honore. 2007. Up- and down-regulation of the mechano-gated K2P channel TREK-1 by PIP2 and other membrane phospholipids. *Pflügers Arch.* 455:97–103. https://doi.org/10.1007/s00424-007-0250-2.

45. Woo, J., Y. K. Jeon, …, S. J. Kim. 2019. Triple arginine residues in the proximal C-terminus of TREK K+ channels are critical for biphasic regulation by phosphatidylinositol 4,5-bisphosphate. *Am. J. Physiol. Physiol.* 316:C312–C324. https://doi.org/10.1152/ajpcell.00417.2018.

46. Woo, J., D. H. Shin, …, S. J. Kim. 2016. Inhibition of TREK-2 K(+) channels by PI(4,5)P2: an intrinsic mode of regulation by intracellular ATP via phosphatidylinositol kinase. *Pflügers Arch.* 468:1389–1402. https://doi.org/10.1007/s00424-016-1847-0.

47. Zhang, Q., J. Fu, …, H. Yang. 2022. ‘C-type’ closed state and gating mechanisms of K2P channels revealed by conformational changes of the TREK-1 channel. *J. Mol. Cell Biol.* 14:mjac002. https://doi.org/10.1093/jmcb/mjac002.

48. Brohawn, S. G., W. Wang, …, R. MacKinnon. 2019. The mechanosensitive ion channel TRAAK is localized to the mammalian node of Ranvier. *Elife.* 8:e50403. https://doi.org/10.7554/elife.50403.

Biophysical Journal, Volume *121*

Supplemental information

Transition between conformational states of the TREK-1 K2P channel promoted by interaction with PIP_2

Adisorn Panasawatwong, Tanadet Pipatpolkai, and Stephen J. Tucker

Supplementary information

Number of molecules in each simulation system

Identification of PIP_2 binding site

Molecules	Up state	Down state
TREK-1	1	1
PC (upper leaflet)	252	252
PC (lower leaflet)	180	192
PS	36	38
PIP_2	24	25
CG-water	10968	11115
Na+	358	365
Cl-	198	198

Potential of mean force during the coarse-grained MD simulation

Molecules	Up state	Down state
TREK-1	1	1
PC (upper leaflet)	252	252
PC (lower leaflet)	236	253
PIP_2	1	1
CG-water	11192	11407
Na^+	207	207
Cl^-	198	198

Identification of PA binding site

Molecules	Up state	Down state
TREK-1	1	1
PC (upper leaflet)	252	252
PC (lower leaflet)	210	230
PA	23	25
CG-water	11458	11423
Na^+	248	252
Cl^-	198	198

Atomistic simulation of TREK-1 structure with PIP_2

Molecules	Up state	Down state
TREK-1	1	1
PC (upper leaflet)	250	250
PC (lower leaflet)	237	249
PIP_2	2	2
TIP3P	11615	11213
K^+	212	212
Cl^-	198	198

Atomistic simulation of TREK-1 structure without PIP_2

Molecules	Up state	Down state
TREK-1	1	1
PC (upper leaflet)	252	252
PC (lower leaflet)	234	256
TIP3P	11458	11471
K^+	202	202
Cl^-	198	198

Figure S1

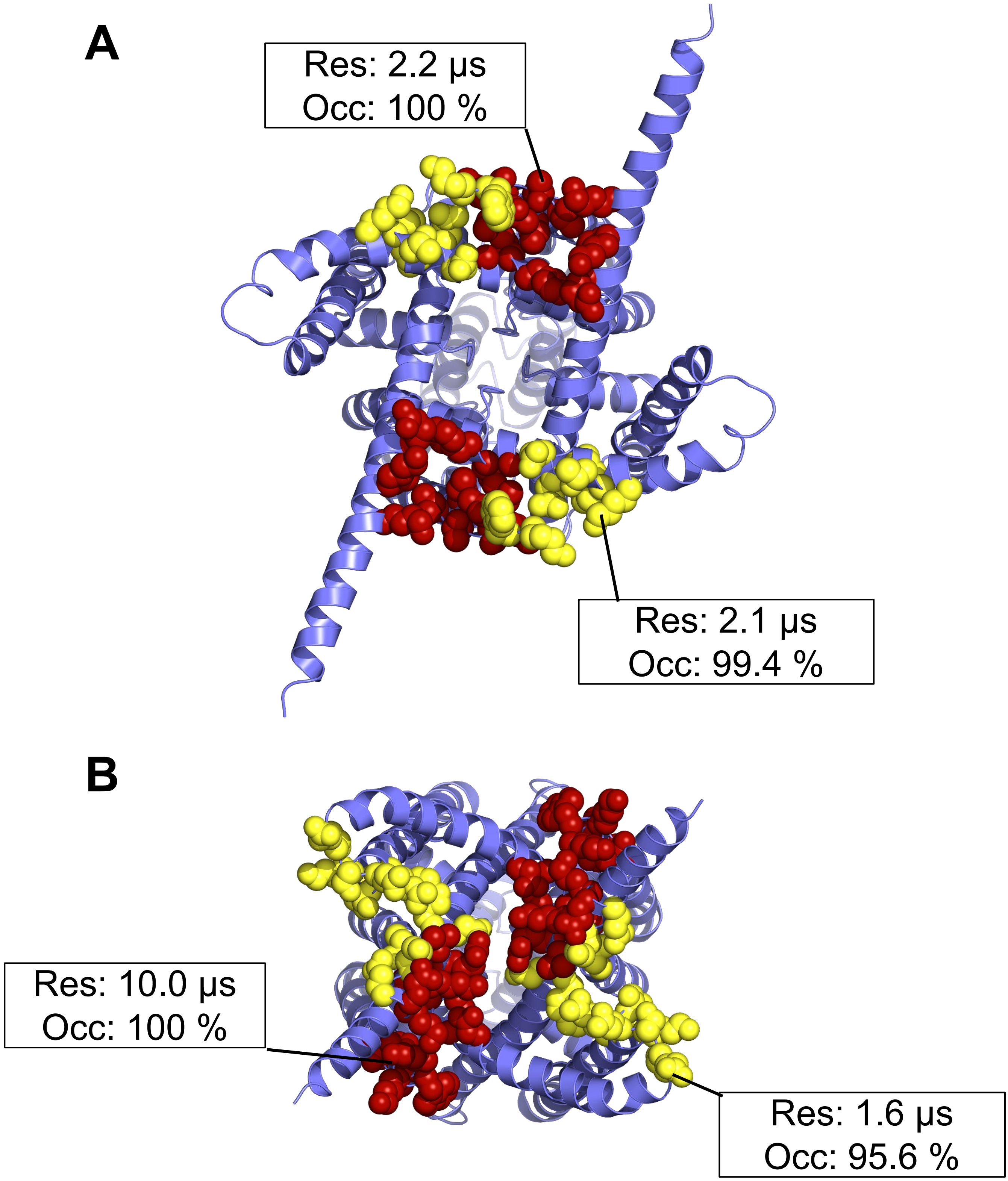

Figure S1. Bottom view of the PIP_2 binding sites from CG simulations

The PIP_2 binding site on the TREK-1 (A) up state or (B) down state. The binding site 1 (red) and 2 (yellow) are shown superimposed relative to their crystal structure. Each site is annotated with their residence time within the binding site (Res) and their occupancy (Occ).

Figure S2

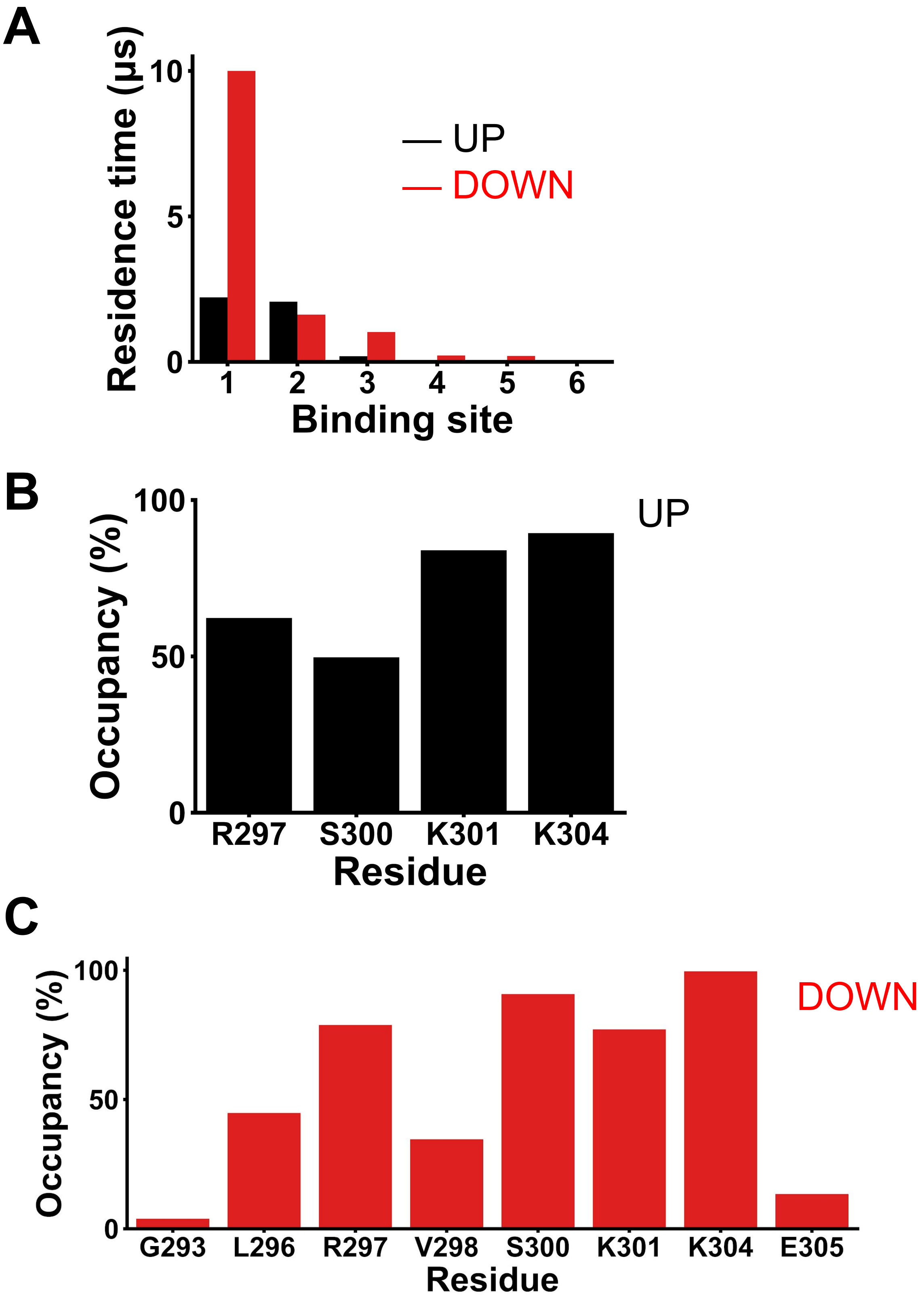

Figure S2. Residence time and occupancy of PIP_2 binding sites from CG simulations.

(**A**) The residence time of PIP_2 in binding sites in the up state (black) and down state (red), ordered by their residence time in the binding site. (**B**) Bar plot of occupancy of residue in the most prominent binding site in the up state (black) and down state (red) from 10 μs CG simulation.

Figure S3

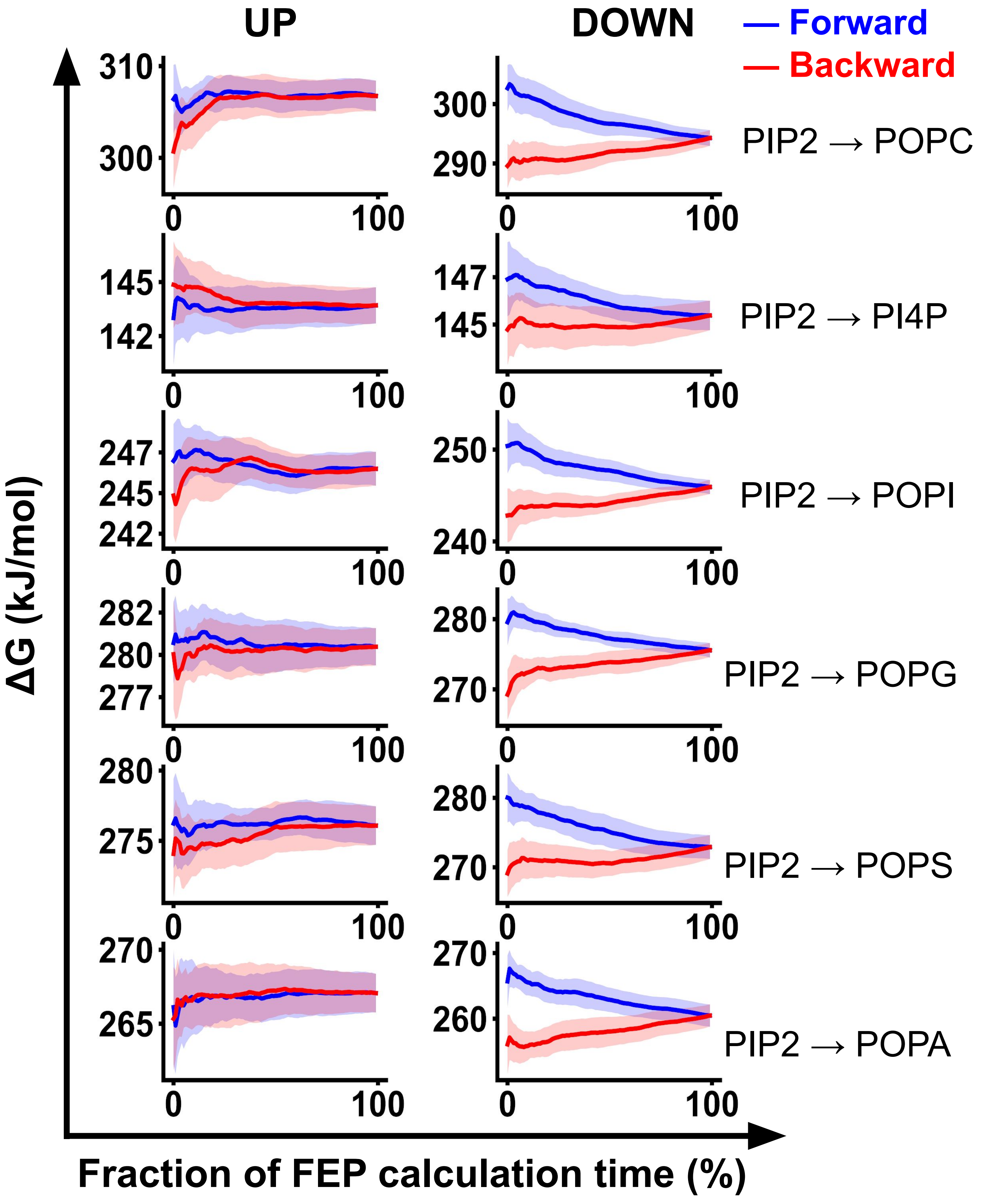

Figure S3. Hysteresis analysis of the free energy calculation whilst PIP_2 is being perturbed to different lipids

Free energy landscape where PIP_2 (0 % of the fraction of FEP calculation time) is perturbed to different lipids (100% of the fraction of the FEP calculation time). The free energy values were calculated from the forward (blue) or backward (red) during the perturbation process. Shaded region represents 95% confidence interval around the mean from 15 repeats.

Figure S4

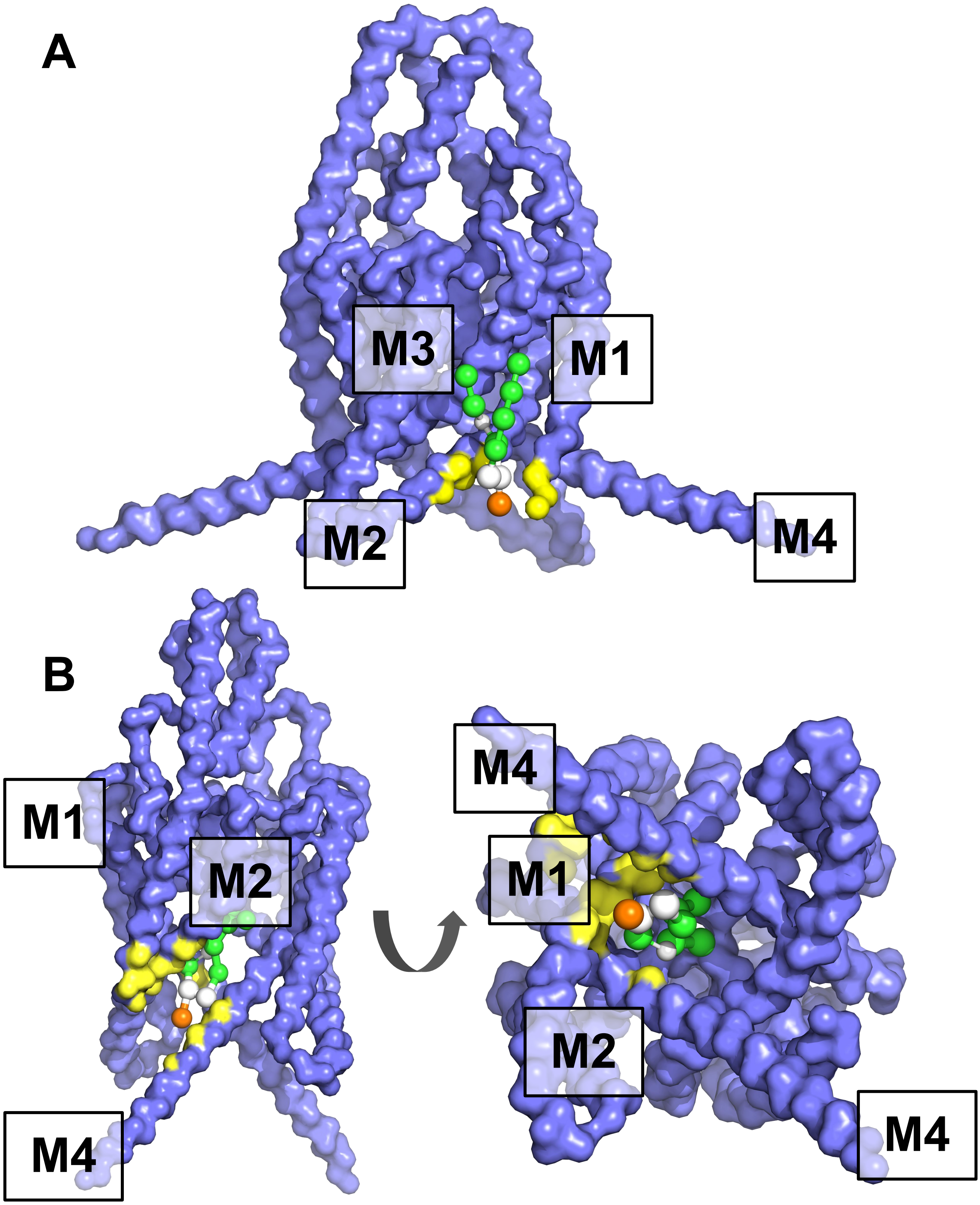

Figure S4. Phosphatidic acid (PA) binding site in the up and down state simulations.

The phosphatidic acid binding sites with highest occupancy and residence time (yellow) are shown superimposing with (**A**) up state and (**B**) down state crystal structures (blue) after a 10μs CG simulation.

Figure S5

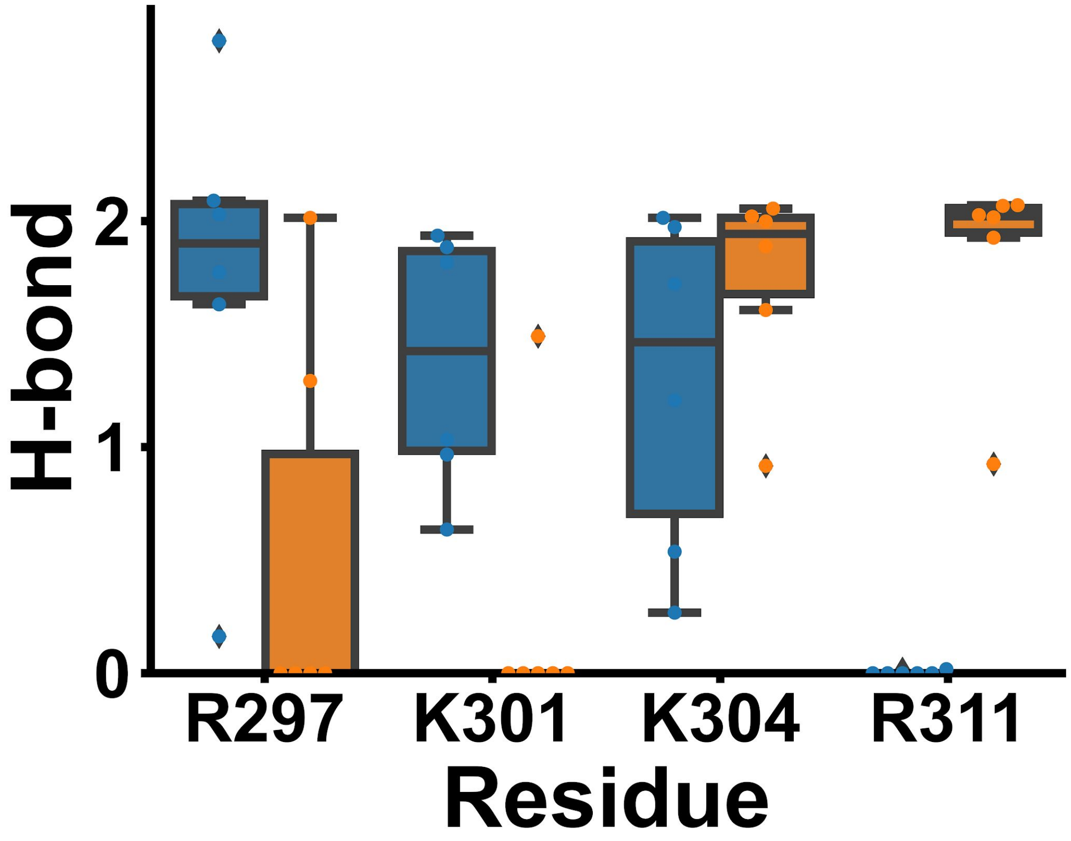

Figure S5. Hydrogen bonding between PIP_2 and TREK-1 in the up and down states

Median number of hydrogen bonds formed from the last 100 ns of the atomistic simulation of TREK-1 in the up state (blue) or down state (orange). Each plot contains six data points from three simulations and each subunit of the TREK-1 channel. The error bar represents the inter-quartile range of the distribution.

Figure S6

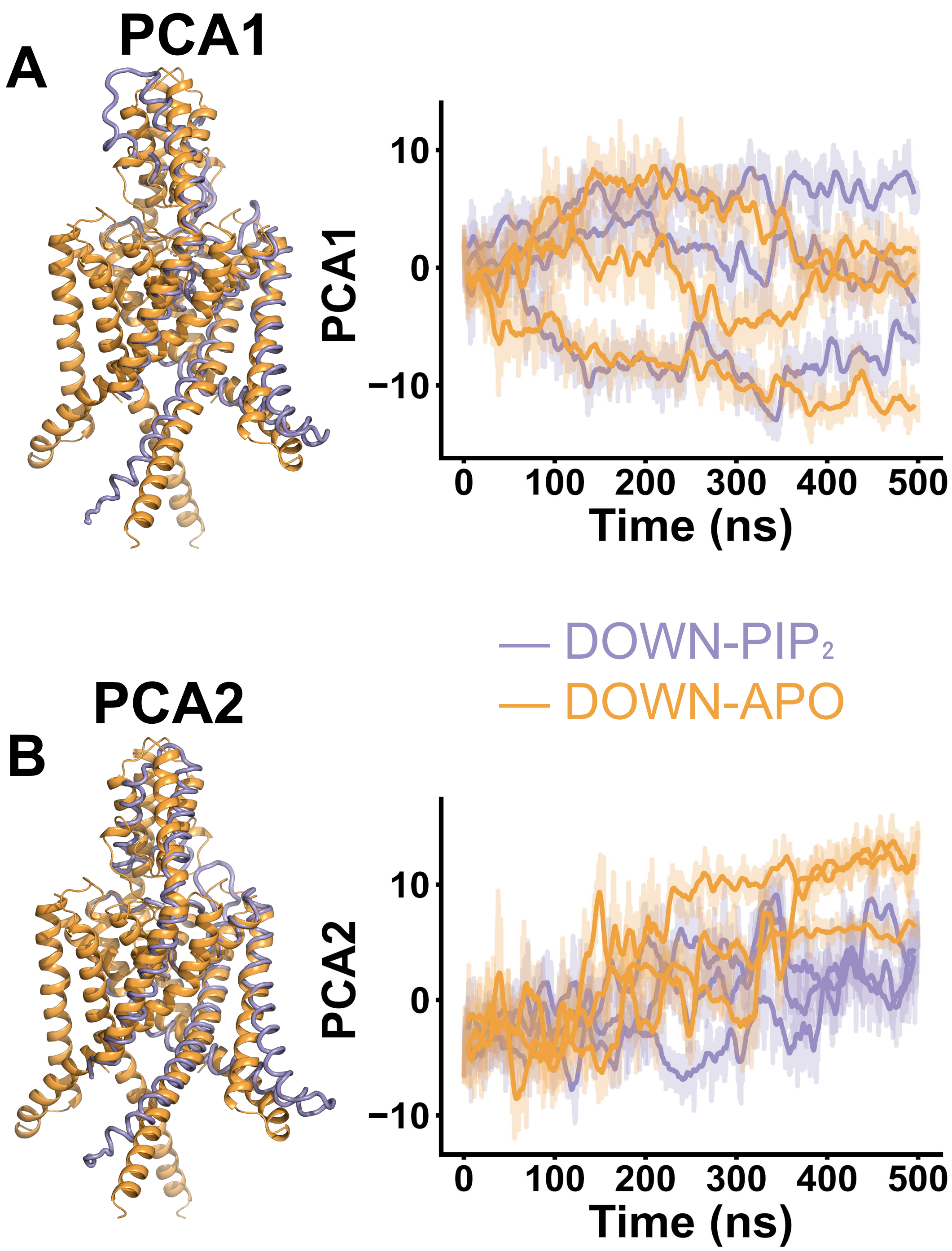

Figure S6. Principal component analysis of PIP2-induced movement in TREK-1

The principal components are calculated from three simulations of TREK-1 channel in the down state containing PIP_2. (**Left**) The backbone of the protein before the simulation (orange) is aligned to the interpolation of the of the most extreme projection along (**A**) the first principle and (**B**) the second principal component (purple) calculated from the simulation. (**Right**) The magnitude of the conformational change along the vector describing the first and second principal component derived from the simulation over 500 ns (n = 3). The simulations were conducted with PIP_2 (purple) and without PIP_2 (orange).

Figure S7

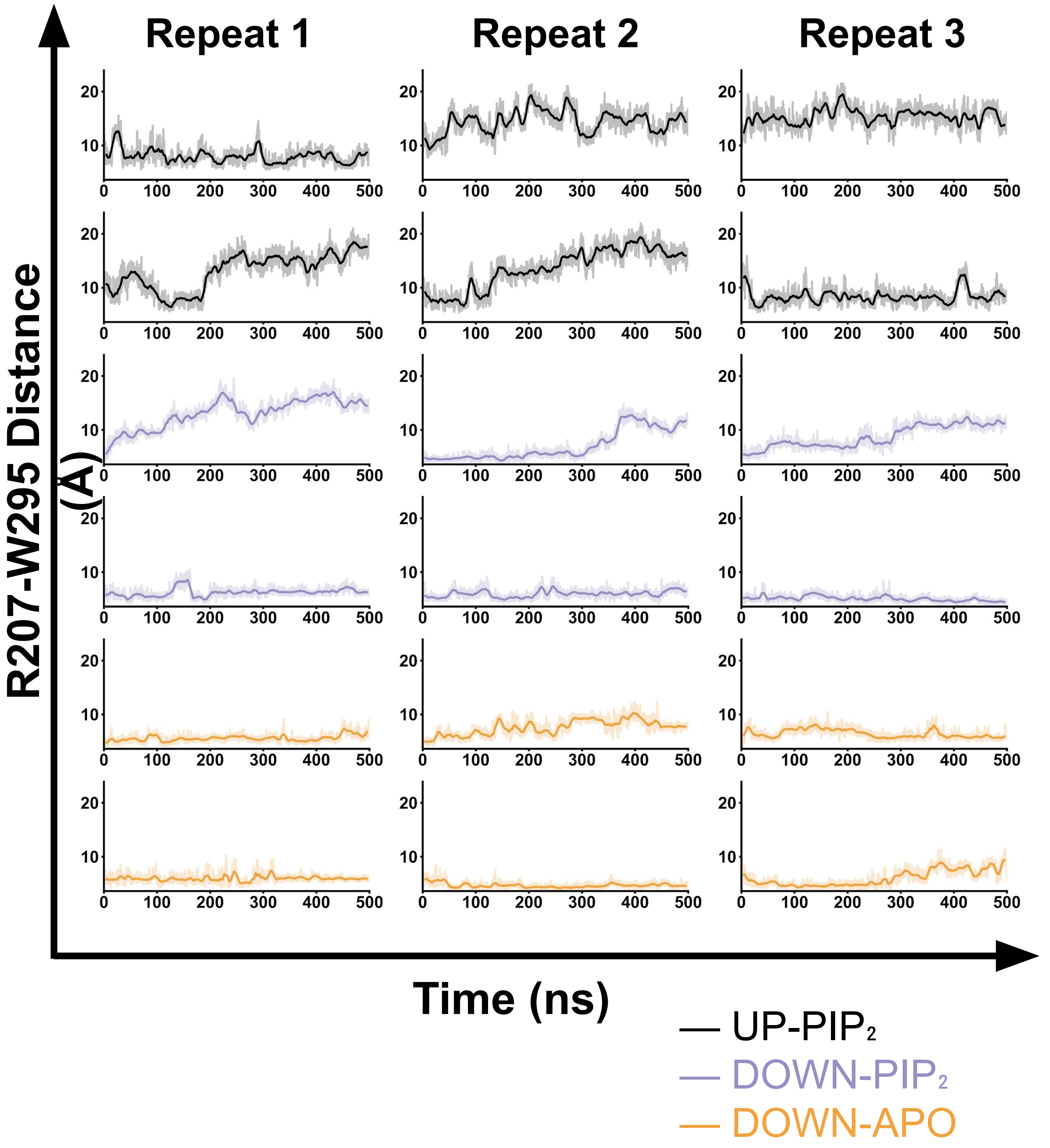

Figure S7. R207-W295 (zipper) distance in all simulations.

Distances between R207-W295 were calculated in three different simulation set-up (up state with PIP_2 - black, Down state with PIP_2 – purple, Down state without PIP_2 - orange). Each measurement represents the distance within each subunit of the TREK-1 channel.

Supplementary files

UP-Visual_Binding_sites.pse

TREK1 channel is represented as cartoon. Two binding sites with highest occupancy are represented in yellow and red spheres.

DOWN-Visual_Binding_sites.pse

TREK1 channel is represented as cartoon. Two binding sites with highest occupancy are represented in yellow and red spheres.
